# A recurrent neural network for soft sensor development using CHO stable pools in fed‐batch process for SARS‐CoV‐2 spike protein production as a vaccine antigen

**DOI:** 10.1002/btpr.70046

**Published:** 2025-06-02

**Authors:** Sebastian‐Juan Reyes, Robert Voyer, Yves Durocher, Olivier Henry, Phuong Lan Pham

**Affiliations:** ^1^ Human Health Therapeutics Research Centre National Research Council Canada Montréal Quebec Canada; ^2^ Department of Chemical Engineering Polytechnique Montreal Montreal Quebec Canada

**Keywords:** CHO fed‐batch bioreactor production, CHO stable pool, data driven sensor, process analytical technologies (PAT), recurrent neural network (RNN), SARS‐CoV‐2 spike protein, soft sensor

## Abstract

Fed‐batch recombinant therapeutic protein (RTP) production processes utilizing Chinese Hamster Ovary (CHO) cells can take a long period of time (>10 days). Within this period, not all critical features may be measured routinely, and in fact, some are only measured once the process is terminated, complicating decision making. As a consequence, utilizing routine current day bioreactor online data to aid in next day predictions is a promising strategy for model predictive control‐based feeding strategies. The article details the development of a proposed soft sensor that merges current day bioreactor online data and offline historical sampling data to generate predictions about the next day of the production process. This approach demonstrated the ability to track product titer, cell growth, key metabolites, and cumulative glucose consumption across the 17‐day process with low normalized root mean squared error (nRMSE = 0.24) and low normalized mean absolute error (nMAE = 0.18) as well as high linearity with respect to ground data (average R^2^ = 0.97). It was also demonstrated that the same model architecture could effectively soft sense product titer and metabolic profiles (glucose, lactate, ammonia) without having sampling day's offline data as inputs to the model. This suggests that the proposed model could act as a true soft sensor of hard‐to‐determine variables such as the trimeric SARS‐CoV‐2 spike protein that relies on end‐of‐process measurements to acquire the data (labor‐intensive semi‐quantitative SDS‐PAGE gels or ELISA assay). Instantaneous specific glucose consumption rates were also predicted and showed good agreement with experimental measurements, further offering opportunities for online glucose control.

## 1 INTRODUCTION

A soft sensor is the concomitant use of software‐implemented models (soft) and hardware devices (sensor) to gather and gain new information about the process.^1^ This is key because without the use of soft sensors, that is to say just exclusively using a sensor, it would be impossible to derive the same information.^2^, ^3^, ^4^, ^5^ At its core, these soft sensors are used with the explicit purpose of leveraging on‐line data in order to infer quantitative information about complex process variables that are impossible to measure directly in a sterile system or can be measured at a very low sampling frequency.^6^ Consequently, soft sensors can become useful tools in terms of monitoring and control applications within the biopharmaceutical industry.^2^, ^3^, ^4^, ^5^ A well‐developed soft sensor that considers the needs of its stakeholders should, in theory, result in a reduction of operational surveillance and maintenance work. Additionally, soft sensors should increase the interpretability of the results of culture runs given the capacity of the models to relate various key variables to each other.^1^ Given this promise, soft sensors are perfect candidates for the PAT initiative to contribute toward automated control.^1^, ^6^ Soft sensors can be split into three categories: model‐driven sensors, data‐driven sensors, and hybrid models.^1^, ^7^

Model‐driven sensors involve mechanistic models that are based on engineering principles, like mass or energy balances. They can provide an understanding of the processes that is inherently ingrained in biological insights.^7^ Such models are capable of introducing known culture conditions such as media composition and/or culture performance indicators (cell growth, titer) to set up tangible models. Because of these characteristics, model‐driven sensors can exploit known kinetic equations that capture dynamic changes of relevant variables.^8^, ^9^ In essence, these soft sensors incorporate reaction kinetics, transport phenomena, and thermodynamic constraints into the model.^8^ However, these types of soft sensors must go through rigorous phases of parameter identification, uncertainty, and sensitivity analysis to properly validate said models. It must be noted that in the case where the model is reproducible and reliable, biologically interpretable information is provided to the end user that can increase the understanding of the production process.^1^, ^8^ Model‐driven soft sensors can be split into two distinct categories: dynamic models and steady‐state models. Dynamic models depend on balances and kinetic presumptions to suitably express rate expressions as functions of the state variables.^10^ On the other hand, steady‐state models originate from mass and heat transfer laws. Good examples of such steady‐state models can be flux balance analysis (FBA) or metabolic flux analysis (MFA) which are stoichiometric‐rooted techniques routinely used to characterize cell metabolism.^11^, ^12^, ^13^ They are also useful in estimating intracellular fluxes by leveraging known extracellular analyte consumption or compound production rates as model constraints. Given that the quasi‐steady state presupposition for intracellular metabolites is key, such models are considered static in nature.^8^ On the other hand, kinetic models are usually expressed as a series of ordinary differential equations (ODEs) that can describe dynamic changes in metabolite concentrations, cell density, and protein expression during the cell culture process.^2^, ^3^, ^4^, ^5^ Because of this, cell growth and cell death can be unambiguously linked to changes in concentration of relevant nutrients and metabolic by‐products. In addition, protein expression has been linked to cell growth and amino acid metabolism.^8^, ^14^, ^15^ As a direct consequence, dynamic models can be designed with varying levels of complexity conditioned on the assumptions made by the researcher regarding the culture system in the bioreactor. This diversified degree of complexity can be tuned by considering heterogeneity within the cell population or by acknowledging the existence of known cellular compartments and their respective behaviors. On the other hand, dynamic models can be simplified if reactions are lumped to rate limiting steps.^8^, ^14^, ^15^ Because of such caveats, model‐driven sensors can be very complex and time consuming to develop.^16^

Data driven soft sensors utilize multivariate data analysis (MVDA) techniques such as partial least squares (PLS), principal component regression (PCR), and non‐linear regressions such as artificial neural networks (ANN) and support vector machine regression (SVMR) in order to relate input features to predict desired variables.^17^, ^18^, ^19^, ^20^, ^21^, ^22^, ^23^, ^24^, ^25^, ^26^, ^27^ These nonlinear models are particularly useful in understanding cell cultures given the fact that a lot of the interactions between key metabolic and process variables remain unknown or are highly cell line specific.^1^ PLS regression and ANN are notably used to analyze spectral data. Under such conditions, the spectral data is used as input and linked to outputs such as substrate concentrations, biomass, cellular viability, or product titer.^28^, ^29^ Thanks to such models, it is possible to predict critical process parameters (CPPs) that are not available through the spectral signals or multi‐sensor data alone but arise from the deconvolution of the datasets generated from such sensors. This, in theory, is an advantage over mechanistic models given that online measurements (temperature, pH, DO, oxygen flowrate, base addition, dissolved carbon dioxide flow rate, oxygen uptake rates, bio‐capacitance signals, Raman spectral data, cell volume) are not directly coupled to cell counts. Metabolic parameters such as lactate production/consumption, ammonia production/accumulation, and glucose consumption can be utilized to help predict said variables (protein expression, cell growth, etc.). Alternative approaches applied in bioprocessing include multiple linear regression, k‐nearest neighbors (KNN), regression trees, ensemble approaches (Gradient Boosting Machine, Extreme Gradient Boosting, Adaptive Boosting, Random Forest) and Gaussian process regression.^30^ Since mammalian cell culture data is complex both in its time‐dependent variation and multivariate nature, methods developed for sequence forecasting have been applied. Even though ANN can capture dynamics of non‐linear systems like cell culture runs, RNN is a subclass of ANN that better captures the internal temporal dependencies of a system. These architectures are particularly useful for making t‐step ahead predictions of relevant state variables.^31^ They have recently been applied in predicting biomass growth before and after transfection of an rAAV production process.^32^ This was done by utilizing cumulative oxygen sparged, dissolved oxygen values, and cumulative dissolved oxygen as features and relating their time‐related variance to cellular growth. A subclass of RNN models denominated long short‐term memory (LSTM) has been applied for multivariate estimation of mammalian cell culture data (total cell density, viable cell density, viability, lactate, glucose, titer) and has also been developed.^33^

Hybrid models, known as gray box models, are another relevant class of soft sensors. These types of soft sensors can be considered to be a combination of data driven soft sensors and mechanistic model driven soft sensors. They have the capacity of utilizing the benefits of each method.^2^, ^3^, ^4^, ^5^, ^9^, ^16^, ^34^, ^35^, ^36^, ^37^, ^38^, ^39^ Various architectural techniques exist when developing hybrid models and they can fall in three general categories: (i) Calibration, (ii) Composition, and (iii) Transformation. Calibration architectures utilize black box models to reduce mechanistic model errors. Composition architectures utilize black box models to estimate unknown terms within a mechanistic model.^16^, ^40^, ^41^ Lastly, a transformation approach utilizes mechanistic models to generate data rich environments from which training a black box model is possible.^42^ Examples of these are state observers that integrate dynamic modeling (white box models) and data driven modeling (black box models). This is realized by updating state estimates derived from noisy measurements and gradually reducing the estimation error with each iteration.^1^ This is usually done assuming linear dynamics within the process and a Gaussian distribution for the error terms. Under such assumptions, a Kalman filter can be used. However, given that the process dynamics within a bioprocess are non‐linear in nature, the extended Kalman filter may be applied. This method realizes a piecewise linearization through a first order Taylor series expansion.^43^ Another important version of the Kalman filter that is widely used for non‐linear systems is the unscented Kalman filter. This method employs an unscented transform to avoid relying on a Taylor series expansion of the system of equations to linearize the model.^44^ This method can be advantageous since the unscented transform allows non linearizable functions to be used as a state observer and thus black box techniques such as support vector machine regression (SVMR) can be utilized so as to relate an online sensor output to a non‐online variable.^44^ It must be noted that because the accuracy of a hybrid soft sensors (gray box models) can be significantly impacted by the accuracy of the mechanistic model embedded within the gray box model, the mechanistic model requires extensive validation to ensure it can successfully represent the process.^45^ Extended Kalman filters have been applied to data generated (cell density, glucose concentration, glutamine concentration, lactate concentration, ammonia concentration, and rAAV viral titer) from HEK293 processes producing recombinant adeno‐associated virus (rAAV), where online viable cell densities (measured through bio‐capacitance signals) and an unstructured mechanistic model are used in conjunction with neural ordinary differential equations (ODEs) to estimate cell‐specific rates. This results in a soft sensor that is able to estimate continuously other state variables that are measured at low frequency.^46^ Likewise, hybrid extended Kalman filters which utilize partial least squares (PLS) in order to estimate specific rates in the model have been developed and applied to CHO fed batch culture datasets (measured variables: viable cell density, the concentration of glucose, lactate, glutamine, ammonium, osmolarity, pH, ${\mathrm{pO}}_{2},{\mathrm{pCO}}_{2}$ and titer).^47^ Hybrid models can also describe the biological system by way of a mechanistic framework but define the cell specific rates through statistical expressions.^2^, ^3^, ^4^, ^5^ The mechanistic framework inherently constrains the solution space of the model and thus, the statistical cell‐specific rate expressions can be automated.^48^ Within this structure, PLS or ANN prediction resulting from multi‐wavelength spectra or multivariate parameters can be fed as inputs into a mechanistic model for CHO fed batch culture monitoring.^49^, ^50^ XGBoost regressors, random forest regressors and multilayer perceptron (MLP) regressors have been used to estimate CHO cell specific rates (specific growth rate, specific productivity, and specific cumulative glucose consumption) throughout a fed‐batch process by utilizing VCD, titer, temperature, glucose concentration, glucose consumed, lactate concentration, and viability as inputs.^41^ These updated specific rates are then utilized as parameters in a mechanistic model to predict relevant culture outcomes (viable cell density, titer, and cumulative glucose consumption).^41^ Hybrid models have also been utilized for the prediction of key product quality attributes (impurity levels, charge variants species, intact mass, total low molecule weight (LMW), and N‐glycan profiling) by coupling a propagation model that describes the time evolution of cell culture variables (viable cell density (VCD), glucose, glutamine, glutamate, lactate, ammonia, cell viability, and titer) with a PLS model that regresses quality attributes as a function of cell culture variables and process conditions.^51^ Alternatively, to increase the overall data richness of a production process, the utilization of unstructured mathematical models would allow the user to generate cell line relevant data so as to create information‐rich environments that can be purposed to train nonlinear deep learning regression techniques like recurrent neural networks (RNN).^52^ It is worth noting that transformer architecture which has been highly successful in natural language processing has been shown to not outperform‐ RNN structures in terms of time series forecasting.^53^, ^54^

In this article, the development of a multivariate long‐term time series forecasting model is detailed. This model is capable of realizing one step ahead predictions of key state variables (titer, viable cell density, cumulative glucose consumption, lactate, ammonia) by relying on both offline sampling data and bioreactor online data. This is key given that the offline sampling data is unevenly spaced with respect to time (every other day or every two days; e.g., total of 17 process days but only 10 measurements) thus, reliance on online data (temperature, pH, base addition, DO, integral of DO, cumulative O_2_ flow, cumulative CO_2_ flow) is required to update predictions on days in which no offline sampling data was available. The model was developed on a dataset generated with multiple cumate inducible CHO‐GS cell stable pools expressing variants of the SARS‐CoV‐2 spike protein with changes in process conditions (cell passage number, MSX supplementation at induction). The important advantage of this data driven method when compared to mechanistic or hybrid modeling is that the fully pre‐trained model can be readily applied by non‐experts as it needs no knowledge about boundary conditions or metabolic networks. This can thus be readily transferred to production processes without additional training on its operators. The model can qualitatively capture the dynamics of metabolic and protein expression profiles by relying exclusively on bioreactor online data and easily accessible viable cell counts throughout the whole 17‐day process. Furthermore, this model advantageously soft senses hard‐to‐measure variables like the SARS‐CoV‐2 spike protein in a daily fashion.

## 2 MATERIALS AND METHODS

### 2.1 Stable CHO Cell Pools and Small‐Scale Cell Culture Conditions

Three stable CHO‐GS cell pools expressing SmT1 trimeric spike proteins, namely Wuhan Tagless (WuTL), Delta (De), and Beta (Be) variant, were generated as described previously.^55^, ^56^, ^57^, ^58^ Stable pool cells were thawed and grown in BalanCD CHO Growth A medium (Fujifilm/Irvine Scientific) supplemented with 50 μM MSX (L‐Methionine Sulfoximine, Sigma‐Aldrich) and 0.1% (w/v) Kolliphor P188 surfactant (Sigma‐Aldrich). 125‐mL (20 mL working volume) shake flasks without baffles (Corning) were used for cell maintenance. The flasks were shaken at 120 rpm (25 mm orbital diameter) in an incubator regulated at 37°C, 5% CO_2_, and 75% relative humidity. Cells were passaged every 2 or 3 days to keep a maximum viable cell density between 2 × 10^6^ and 3 × 10^6^ cells/mL.

### 2.2 Cell Culture Analytical Methods

Viable and total cell density, cell viability, main metabolites (glucose, lactate, ammonia) were measured utilizing the previously reported methodology.^55^, ^56^, ^57^, ^58^ Briefly, cell counts were performed with Innovatis Cedex (Roche) or ViCell Blue (Beckman Coulter) automated cell counters using trypan blue dye exclusion assay. Key metabolites such as glucose, lactate, and ammonia were determined using the Vitros 350 Chemistry System (Orthoclinical Diagnostics). Volumetric protein titers were estimated using TGX Stain‐free SDS‐PAGE gels (Bio‐Rad) quantification method. Table 1 summarizes online and offline measurements as well as the respective relative standard deviations for analytical measurements, which were measured from replicate measurements using platform data.

**TABLE 1:** Process variables and parameters (pH, temperature and DO) considered in the model and relative standard deviations in analytical measurements.

Offline Measurements	Cell Growth	VCD (cells/mL)	7%
	Metabolites	Lactate (mM)	2%
		Ammonia (mM)	3%
		Cumulative Glucose Consumed (mM)	3%
	Protein Production	Titer (mg/L)	12%
Online Continuous Measurements	pH Control	pH profile	‐
		Base Addition Volume (mL)	‐
		Total Carbon Dioxide sparged (mL)	‐
	Oxygen Requirements	Total Oxygen sparged (mL)	‐
		DO (% of air saturation)	‐
		Integral DO (%*Day)	‐
	Temperature Control	Temperature (°C)	‐

### 2.3 Fed‐batch Cell Culture Process Conditions

All productions were conducted in parallel benchtop bioreactors 0.75 L Multifors 2 (Infors) under the conditions detailed in Table 2. Corning shake flasks were used to generate the seed trains. The bioreactors were seeded at 0.4 × 10^6^ cells/mL and cultivated for 17 days. Temperature downshift (37°C to 32°C) was realized 3 days after seeding. A pH shift was conducted 2 days post‐seeding (from 7.05 ± 0.05 to 6.95 ± 0.05). A dissolved oxygen (DO) set point of 40% (of air saturation) was chosen. Micro‐spargers with a 0.0033 vvm (volume of gas per initial working volume per minute) air cap were implemented in a cascade air/oxygen strategy. Air flow rate was automatically increased to a selected maximum value (air cap) then remained constant to the end. Pure oxygen was injected as needed to maintain the DO setpoint. CO_2_ and an in‐house mix of NaHCO_3_/NaOH were used to maintain pH within its selected deadband. Production induction was initiated with the addition of 4‐Isopropylbenzenecarboxylate (Cumate, ArkPham). Cultures were fed with BalanCD CHO Feed 4 (Fujifilm/Irvine Scientific) and supplemented with glucose as needed to maintain glucose concentration above 17 mM (3.06 g/L) for the next sampling point. Glucose supplementation relied on estimating glucose consumption between sampling days and extrapolating the observed glucose consumption for the next sampling period. Consequently, enough glucose is added to counteract the expected glucose consumption such that the residual glucose is maintained above 17 mM. Samples were taken from the bioreactors on days −3, −2, −1, 0, 3, 5, 7, 10, 12, and 14 dpi (days post‐induction) for off‐line analysis, while feeding was realized in a bolus dosage from 0 dpi (induction day) onward. Cell passage number was varied across different batches (passage 5, 8, and 11) to study the impact of cell age on pool expression stability. It is known that the cell pool is heterogeneous, composing of different cells with different expression levels in contrast to a stable clone.^59^ Therefore, it is critical to determine the cell age operation window to avoid a significant expression loss when the cell passage number increases.^56^ Additionally, MSX supplementation (75 μM) at induction (0 dpi) was also investigated to evaluate the impact of high MSX concentration on production performance. High MSX concentration has enabled increased protein expression observed in our previous unpublished data; this effect was also shown with another group.^60^ Volumetric power input (P/V) indicating the relationship between agitation speed and culture volume, was set in a range between 40 and 30 W/m^3^ as it decreased with every feed bolus addition. Raw data of all the culture conditions can be visualized in Figures S1–S5 of Annex.

**TABLE 2:** Bioreactor production process conditions.

Pool	Seeding Density (10^6^ cells/mL)	Cell Passage Number	MSX (μM)	P/V Range (W/m^3^)	DO (%)	Kolliphor P188 (%, w/v)	Sparger	Aeration Strategy	Number of Impellers	Feed 4 Supplementation (% based on initial volume)
Delta	0.4	5 8 11	50 125	40–30	40	0.2	Micro	Air cap (AC) with Air/O_2_ cascade	2	0 dpi (5%), 3 dpi (5%), 5 dpi (5%), 7 dpi (7.5%), 10 dpi (5%), 12 dpi (5%)
Beta	0.4	5 8 11	50 125	40–30	40	0.2	Macro	Air cap (AC) with Air/O_2_ cascade	2	0 dpi (5%), 3 dpi (5%), 5 dpi (5%), 7 dpi (7.5%), 10 dpi (5%), 12 dpi (5%)
WuhanTL (WuTL)	0.4	5 8 11	50 125	40–30	40	0.2	Macro	Air cap (AC) with Air/O_2_ cascade	2	0 dpi (5%), 3 dpi (5%), 5 dpi (5%), 7 dpi (7.5%), 10 dpi (5%), 12 dpi (5%)

### 2.4 Dataset and data handling methodology

The dataset is made up of 21 production runs. 10 runs were performed with the Delta pool (De), 6 runs with Beta pool (Be), and 5 runs with the Wuhan Tag‐less pool (Wu‐TL). For all the productions, viable cell density, cumulative glucose consumption, lactate, ammonia, and titer were measured or calculated. Cumulative glucose consumption was estimated by adding up the glucose consumed between sampling days (difference between media glucose concentration after feed and measured glucose concentration in next sampling day) thus keeping track of total glucose consumption. Online data underwent a structured preprocessing workflow. First, Savitzky–Golay filtering was applied to the pH data to reduce sensor noise.^59^ Next, daily averages were calculated for online pH, DO, and temperature measurements to smooth temporal fluctuations.^59^ For other variables, such as base addition, oxygen, and carbon dioxide sparging, root cause analysis was employed to identify and exclude data points influenced by sensor faults or anomalies. As a result, key variables including DO, the integral of DO (${\mathrm{DO}}_{\mathrm{int}}$), total oxygen sparged, pH, base addition, total carbon dioxide sparged, and temperature were compiled into Excel spreadsheets (Microsoft), allowing for direct comparison with sampling day data. Integral of the DO curve (${\mathrm{DO}}_{\mathrm{int}}$) was chosen as a variable to aid in giving the model information about possible changes in the DO profile between monitoring days. Integral of *DO*, $O_{2}$ sparge rates, and ${\mathrm{CO}}_{2}$ sparge rates were estimated by calculating the area under the curve of each signal through trapezoidal rule for numerical integration:

$${\mathrm{DO}}_{\mathrm{int}}=\sum_{i=1}^{n}\frac{\left({\text{time}}_{i+1}-{\text{time}}_{i}\right)\times \left({\mathrm{DO}}_{i+1}+{\mathrm{DO}}_{i}\right)}{2}$$

$$\text{Total Oxygen sparged}=\sum_{i=1}^{n}\frac{\left({\text{time}}_{i+1}-{\text{time}}_{i}\right)\times \left(O_{2{\_}_{i+1}}+O_{2\_i}\right)}{2}$$

$$\text{Total Carbon Dioxide sparged}=\sum_{i=1}^{n}\frac{\left({\text{time}}_{i+1}-{\text{time}}_{i}\right)\times \left({\mathrm{CO}}_{2_{\_i+1}}+{\mathrm{CO}}_{2\_i}\right)}{2}$$

where *i* is the counter that ranges from *1* to *n* (from the first data point until the end of the data in the time series); ${\text{time}}_{i}$ represents the time associated with the *i*th data point; ${\text{time}}_{i+1}$ is the time associated with the *(i + 1)*th data point; ${\mathrm{DO}}_{i}$ represents the value of DO at the *i*th data point; ${\mathrm{DO}}_{i+1}$ represents the value of DO at the *(i + 1)*th. Similarly, $O_{2\_i}$ and ${\mathrm{CO}}_{2\_i}$ represent the respective gas flow value at the *i*th data point, while $O_{2{\_}_{i}+1}$ and ${\mathrm{CO}}_{2{\_}_{i}+1}$ signify the *(i + 1)*th data point of the respective gas flows.

### 2.5 Details of the RNN methodology

**FIGURE 1:**
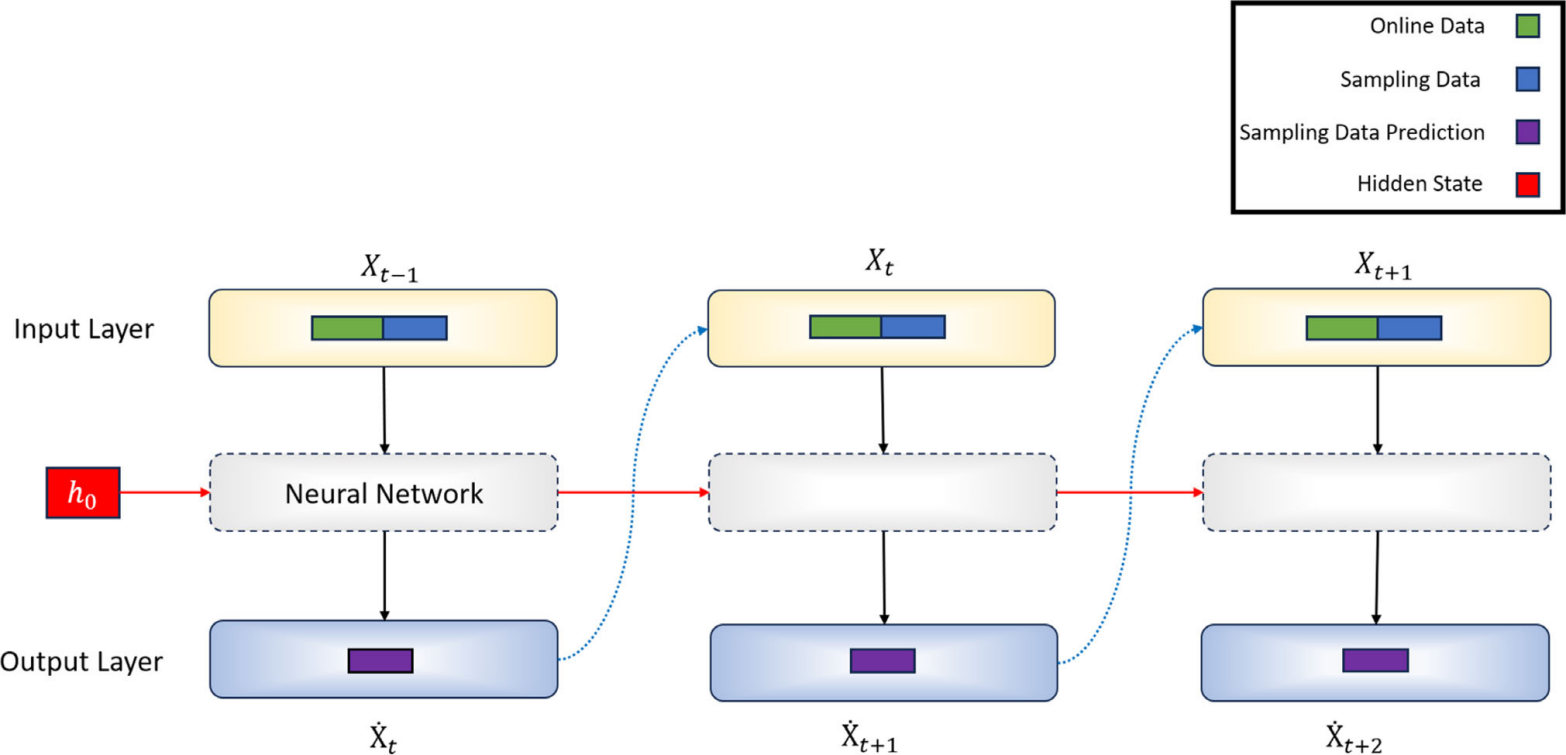
Soft sensor architecture for predicting next day sampling data. Online bioreactor data (total oxygen sparged, total carbon dioxide sparged, pH, base addition, DO, integral of DO, temperature) and measured sampling data (lactate, ammonia, cumulative consumed glucose, viable cell density, titer) along with an initial hidden state are received as inputs to a neural network. This neural network outputs the next discrete time prediction of each sampled data along with an updated hidden state to be used in the next iteration. This process is repeated for all days during the production process (17 days). If there is no available sampling data, then the bioreactor online data and the previous output predictions (rather than the ground sampling data) will be used as inputs in the next iteration.

Recurrent neural networks (RNN) are a family of neural networks for processing sequential data. There is a shared similarity with multilayer perceptron (MLP). The general network structure is represented by an input layer, one (or numerous) hidden layers, and an output layer. However, the RNN structure (Figure 1) allows the model to carry over latent information from time step to time step, thus capturing time‐varying profiles.^61^ RNNs and MLPs have distinct differences that have significant implications for sequence learning. While MLPs can only map from input to output vectors, RNNs have the ability to map from the entire history of previous inputs to each output. This means that an RNN can leverage a memory of past inputs stored in its internal state to influence the network's output.^62^ In fact, the universal approximation theory applies to both RNNs and MLPs, but with different implications. For MLPs, it states that with enough hidden units, an MLP can approximate any measurable mapping from input to output. On the other hand, for RNNs, the equivalent result is that with a sufficient number of hidden units, an RNN can approximate any measurable sequence‐to‐sequence mapping.^62^ RNNs are specifically designed for processing sequential data, just as convolutional neural networks (CNNs) are specialized for processing grid‐like data such as images.^63^ RNNs excel at handling sequences of values, and they can scale to longer sequences compared to networks without sequence‐based specialization. Additionally, most RNNs are capable of processing sequences of variable length, providing flexibility in handling diverse data inputs.^63^

The latent information, which functions as a network memory, is captured within the hidden state ($h_{t}$), which is updated at each iteration as described in the equations below:

$${\mathrm{MLP}}_{\text{hidden}}:h_{t}=W_{\text{hidden}}\times \Phi \left(W_{\text{in}}\times \left[h_{t-1},O_{t},X_{t}\right]\right)$$

$${\mathrm{MLP}}_{\text{sampled}}^{i}:{Ẋ}_{t+1}^{i}=W_{\text{out}}^{i}\times \Phi \left(W_{h\_\text{in}}^{i}\times h_{t}\right)$$

${\mathrm{MLP}}_{\text{hidden}}$ is multilayer perceptron that receives sampled data and online data, *t* – discrete time index, $h_{t}$ – hidden state at time step *t*, $W_{\text{hidden}}$ – hidden state weight matrix, Φ – standard hidden layer activation function in ANN (logistic, hyperbolic, tangent, sigmoidal, etc.), $W_{\text{in}}$ – input weight matrix, $h_{t-1}$ – hidden state at time step *t‐1*, $O_{t}$ – online variable at time t $,X_{t}$ – sampled input vector at time t; ${\mathrm{MLP}}_{\text{sampled}}^{i}$ – multilayer perceptron that projects the resulting hidden state to a sampled variable space and predicts the updated time series prediction for all *i* sampled variables (titer, viable cell density, lactate, ammonia, cumulative glucose consumption), ${Ẋ}_{t+1}^{i}$ – state vector predictions at a future time discrete index for all *i* sampled inputs (titer, viable cell density, lactate, ammonia, cumulative glucose consumption); $W_{\text{out}}^{i}$ – intermediate hidden state to predicted variable weight matrix for all *i* sampled variables, Φ – standard hidden layer activation function in ANN (logistic, hyperbolic, tangent, sigmoidal, etc.), $W_{h\_\text{in}}^{i}$ – weight matrix to transition from a global hidden state to a variable specific hidden state for all *i* sampled variables, $h_{t}$ – hidden state at time step *t*.^31^, ^33^

**FIGURE 2:**
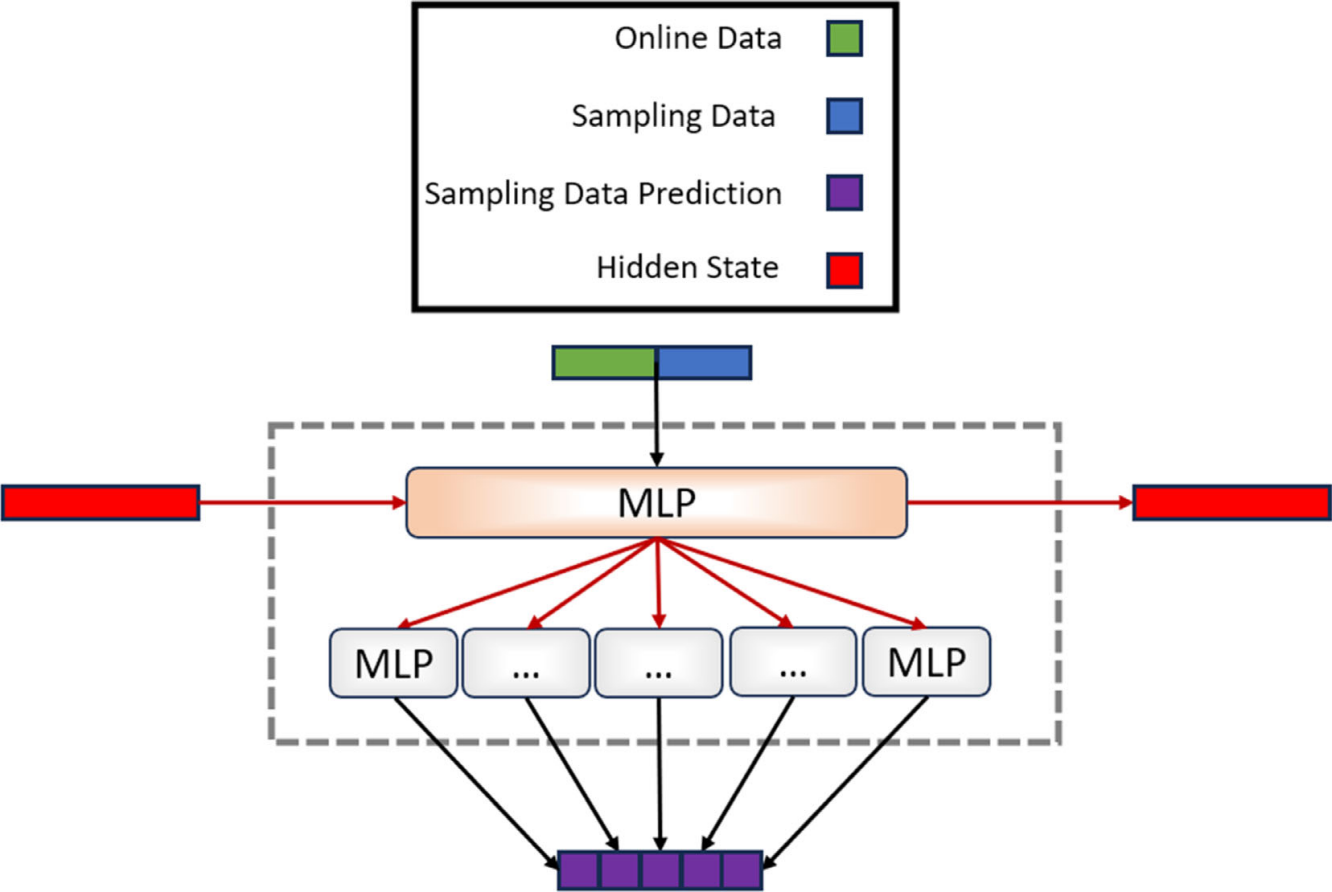
Internal neural network architecture. Offline sampling data, bioreactor online data, and hidden state values are received as inputs to an MLP. The MLP outputs an updated hidden state which serves as input for several MLPs (an MLP for each measured variable). Each MLP projects the resulting hidden state to a sampled variable space and predicts the updated time series prediction for each variable. The hidden state is stored for the next iteration.

For the purpose of this paper, the rectified linear unit (ReLu) function was utilized. The hidden state serves as an internal representation of the network and captures information about previous inputs in the sequence. The hidden state is updated at each time step and serves as a way for the network to maintain information about the context or history of the input sequence (Figure 2). The initial hidden state *h0* was initialized with a zero vector, which can be interpreted as carrying over no information from the past, as the process had not yet been initialized.

Data preprocessing was done with Pandas and Numpy which are important libraries of Python (version 3.9.13) programming.^64^, ^65^ Architecture design and training of the soft sensor were done with PyTorch.^66^ The following hyperparameters were used: The MLPs were all conformed by 256 hidden units. All MLPs utilized a rectified linear unit activation function (ReLu). Learning rate was set to 1.6e‐4. All input data were mean centered and standardized for each variable. Stochastic gradient descent (SGD) was employed as the optimizer with momentum of 0.97 and weight decay of 0.125 to avoid over fitting in the training phase. The training phase included 5000 epochs. An 80% training and 20% test split on the dataset was approximated. Training‐test split was randomly realized such that 8 Delta cultures are used to train the network while 2 are left for testing. Similarly, 5 Beta cultures were used to train and 1 was left over to test. Lastly, 4 Wuhan Tag‐less cultures were used to train and 1 culture was left over to test. Regarding model validation, root mean squared error (RMSE), mean absolute error (MAE) and coefficient of determination ($R^{2}$) were used as validation metrics to evaluate model performance for each predicted feature (e.g., glucose consumed per day, lactate, ammonia, viable cell density, titer) across individual culture run. RMSE, MAE, and $R^{2}$ were first calculated for each batch prediction then averaged out across batches. To facilitate comparisons across features, RMSE and MAE values for each feature in every batch were normalized by the corresponding standard deviation of the feature in that batch. This normalization yields unbiased estimates, normalized RMSE (nRMSE) and normalized MAE (nMAE). Having normalized errors (nMAE and nRMSE) below 1 indicates that the model's prediction errors are smaller than the inherent variability in the experimental data, as represented by the feature's standard deviation within the experimental runs.^32^, ^33^

$${\text{RMSE}}_{\text{global}}=\frac{\sum \sqrt{\frac{1}{n}\times \sum_{i=1}^{n}{\left(y_{\text{ground}\_\text{truth}}-y_{\text{pred}}\right)}^{2}}}{\text{number of batches}}$$

$${\mathrm{MAE}}_{\text{global}}=\frac{\sum \frac{1}{n}*\sum_{i=1}^{n}\mathrm{abs}\left(y_{\text{ground}\_\text{truth}}-y_{\text{pred}}\right)}{\text{number of batches}}$$

$${\text{nRMSE}}_{\text{global}}=\frac{\sum \frac{\sqrt{\frac{1}{n}*\sum_{i=1}^{n}{\left(y_{\text{ground}\_\text{truth}}-y_{\text{pred}}\right)}^{2}}}{\text{Standard}\_\text{Deviation}}}{\text{number of batches}}$$

$${\text{nMAE}}_{\text{global}}=\frac{\sum \frac{\frac{1}{n}*\sum_{i=1}^{n}\mathrm{abs}\left(y_{\text{ground}\_\text{truth}}-y_{\text{pred}}\right)}{\text{Standard}\_\text{Deviation}}}{\text{number of batches}}$$

Here, $y_{\text{ground}\_\text{truth}}$ is the real sampled data of a feature, $y_{\text{pred}}$ is the prediction of a feature from the model, i represents the *i*th value of a given feature in a batch, and n represents the total number of values of a feature in a batch, Standard_Deviation is the standard deviation of a batch for a given feature. Calculating nRMSE and nMAE for the test set is particularly important; values significantly below 1 demonstrate that the model achieves higher precision than randomly selecting parameter values from the experimental ensemble distribution.^33^ Additionally, estimating error metrics for each unique batch allowed for the calculation of statistical measures such as the mean and standard deviation of model errors, providing a comprehensive view of model performance.

## 3 RESULTS AND DISCUSSION

**FIGURE 3:**
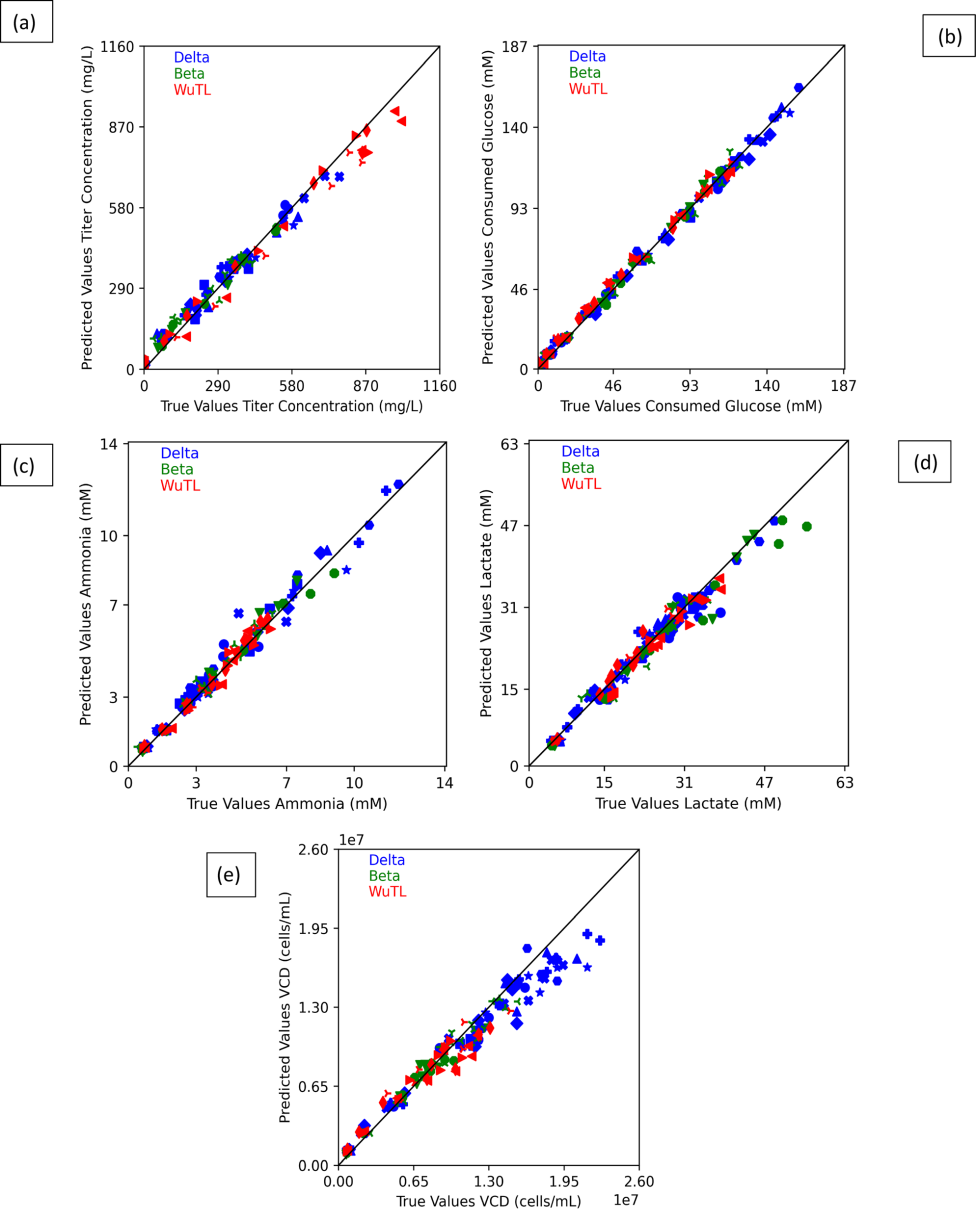
Model results for trained features in the training dataset comprised of 17 cultures (8 Delta pool batches, 5 Beta pool batches and 4 WuTL pool batches). Scatter plots of every batch prediction versus every batch sampled data for the whole training set. Diagonal line represents the $R^{2}$ = 1 ideal model. (a) Titer. (b) Cumulative glucose consumed. (c) Ammonia. (d) Lactate. (e) Viable cell density (VCD). Pools are color coded; Beta is blue, delta is green and WuTL is red. Each unique symbol represents an individual batch culture. There are 9 comparable data points for each culture which results in 153 points (17 × 9) on each graph.

The prediction versus ground truth sampled data scatter plot for each of the 17 training batches is shown in Figure 3. A clear linear relationship ($R^{2}$ values above 0.95 as indicated in Table 3) is seen in all feature predictions (titer, cumulative consumed glucose, ammonia, lactate, and VCD). The scatter plot results can center around the diagonal line which represents the $R^{2}$ = 1 ideal model. It must be noted that for lactate and VCD predictions (Figure 3d,e), there exists a slight deviation from the $R^{2}$ = 1 at higher concentrations. This dataset focused on evaluating the effects of increased cell age on protein production and determining whether higher MSX concentrations (from 50 μM to 125 μM) at induction could enhance protein production outcomes.^59^ Both the WuTL and Beta pools showed stable protein production across different passage numbers. In contrast, for the Delta pool, passage number influenced the variability of key metrics, with higher passage numbers resulting in greater spread in the data. Average protein concentrations decreased with higher passage numbers (P5: 600 mg/L, P8: 361 mg/L, P11: 398 mg/L), while ammonia accumulation increased with cell age, particularly at P8 and P11.^59^ No significant differences were observed between the base (50 μM MSX) and higher MSX (125 μM MSX) conditions, and accounting for passage number revealed that MSX supplementation did not significantly affect cell behavior.^59^ Importantly, each CHO pool exhibited distinct growth patterns and protein production characteristics, with the Delta pool showing the highest VCD accumulation and the WuTL pool achieving higher average spike protein yield (Figures S1–S5). The Delta pool dataset exhibited the most variability, particularly in terms of lactate and ammonia accumulation (Figures S1–S5). which again could be related to its pool age related impacts.

**TABLE 3:** Global RMSE, MAE, nRMSE, nMAE, $R^{2}$ metrics for titer, ammonia, cGC (cumulative glucose consumed), lactate, VCD (viable cell density) for train and test datasets and their respective standard deviations.

	${\text{RMSE}}_{\text{train}}$	${\mathrm{MAE}}_{\text{train}}$	${R^{2}}_{\text{train}}$	${\text{nMAE}}_{\text{train}}$	${\text{nRMSE}}_{\text{train}}$	$\sigma {\text{RMSE}}_{\text{train}}$	$\sigma {\mathrm{MAE}}_{\text{train}}$	${\text{RMSE}}_{\text{test}}$	${\mathrm{MAE}}_{\text{test}}$	${R^{2}}_{\text{test}}$	${\text{nMAE}}_{\text{test}}$	${\text{nRMSE}}_{\text{test}}$	$\sigma {\text{RMSE}}_{\text{test}}$	$\sigma {\mathrm{MAE}}_{\text{test}}$
**Titer**	33.5	27.7	0.99	0.16	0.13	10.3	8.3	51.5	38.8	0.96	0.18	0.24	23.0	15.6
**Ammonia**	0.4	0.3	0.97	0.18	0.14	0.1	0.1	0.4	0.3	0.98	0.12	0.18	0.1	0.1
**cGC**	3.3	2.8	1.00	0.08	0.06	0.7	0.7	7.9	6.2	0.99	0.16	0.20	4.4	3.3
**Lactate**	2.0	1.5	0.97	0.23	0.17	0.8	0.5	2.8	2.3	0.94	0.25	0.31	1.1	0.9
**VCD**	1.24e6	9.74e5	0.97	0.24	0.19	5.02e5	3.68e5	1.36e6.	1.03e6	0.96	0.20	0.26	7.03e5	5.49e5

The time series progression of each feature in the training set (Figures S6–S10) demonstrates that the span of predictions closely aligns with the span of the true data for all pool types. This alignment indicates that the training phase effectively captured the distinct growth and metabolic characteristics unique to each cell pool. Additionally, an examination of the percentage error time profiles reveals a clear pattern: errors decrease significantly after the initial two predictions and subsequently stabilize at values below 20% across most features. This consistency suggests that model accuracy remains robust in the training phase throughout the 17‐day prediction process without noticeable degradation over time.

**FIGURE 4:**
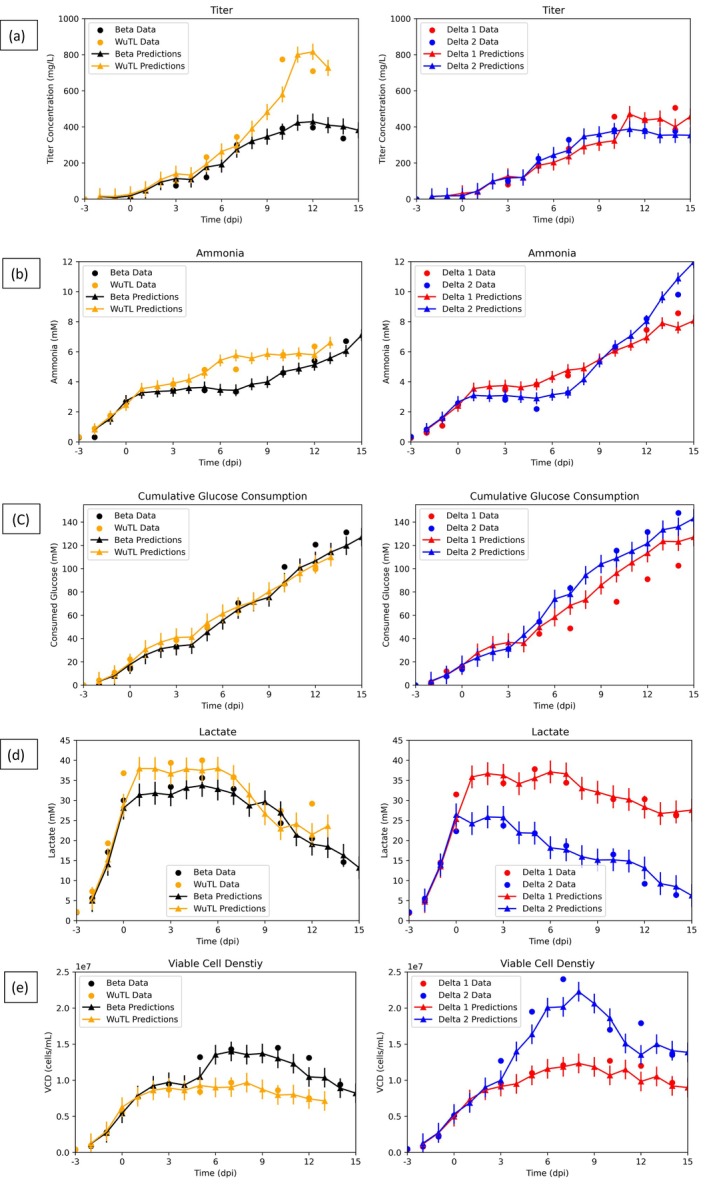
Model results for key features in the test dataset comprised of 4 cultures (1 Beta pool batch, 1 WuTL pool batch and 2 Delta pool batches). Continuous lines with triangle dots are everyday predictions, while circular dots are measured values. (a) Titer profile. (b) Ammonia concentration. (c) Cumulative glucose consumption. (d) Lactate concentration. (e) Viable cell density. The error bars represent the global root‐mean‐square errors of the RNN predictions based on the test dataset and the corresponding target variable.

Next, to determine if the model can generalize, it was tested on 4 production runs that were not utilized in the training process. These hold‐over cultures consisted of 2 Delta cultures, 1 Beta culture, and 1 WuTL culture (Figure 4). The WuTL production was terminated 2 sampling days earlier than normal (harvest at 12 dpi rather than 14 dpi). As it can be seen from Figure 4, time profile tracking of key features is possible even in non‐sampling days. This is key given the fact that the manufacturing process lasts 17 days in which it is impractical to have every day sampled values, especially for the long‐to‐measure outputs such as protein determined by SDS‐PAGE, which is the case for SARS‐CoV‐2 spike protein. For these non‐sampling days, predictions are carried out by relying on previous day model predictions and the online data of the bioreactor (base addition, oxygen flow, DO, integral of DO, pH, temperature). In Figure 4a, it is clear that titer time series tracking is possible despite differences in pool protein production behavior. For example, the Beta and WuTL pools demonstrate vastly different endpoint titer results despite having identical kinetics from 0 to 7 dpi (Figure 4a, left side). Both Delta pools demonstrate similar protein production behavior until 9 dpi, from which Delta 1 culture outperforms Delta 2 culture by close to 100 mg/L (Figure 4a, right side). This subtle increase in the end phase of the culture is also captured by the model, although global RMSE overlaps between both cultures. From Figure 4b, ammonia profiles can be detailed. It is clear that the Beta and WuTL pools exhibit different ammonia accumulation kinetics after the onset of protein production (0 dpi). Increased ammonia accumulation is evident in the WuTL culture following the onset of induction while the Beta pool has an accumulation lag between 0 and 7 dpi, followed by rapid ammonia accumulation after 7 dpi. These changes in kinetics are predicted by the model despite having no measured sampling values at the points in which the kinetics begin to diverge. Similarly, for the Delta cultures, both productions show a plateau in ammonia accumulation from 0 to 7 dpi and then an increase accumulation until 14 dpi (Figure 4b, right side). This behavior is tracked by the model even between 7 to 10 dpi, in which no sampling data exist. From Figure 4c, it can be seen how the cumulative consumed glucose profile changes with culture time, namely the deceleration in cumulative consumption following the temperature shift from 37°C to 32°C. This reduction in glucose consumption can be linked to the cell growth slowdown that occurred at a lower temperature. Delta 1 pool predictions overestimate (from 5dpi onwards) the total profile such that endpoint cumulative glucose consumed is overestimated by 20.5 mM. This may be caused by the fact that Delta 1 culture has high lactate accumulation (above 30 mM) (Figure 4d) and thus the model may be predicting the cumulative glucose consumption to be representatively higher. The Beta culture glucose consumed is underestimated from 10 dpi until the end of the process, such that the measured values do not overlap with the global RMSE of model predictions. Figure 4d shows how lactate profiles can be tracked with the proposed model. For the Delta cultures, large differences in peak lactate values are observed which, in turn, impacts the lactate re‐absorption profile (5–14 dpi). These differences in kinetics for the same pool in two different culture runs are captured by the model. Moreover, it is worth mentioning that Delta 1 culture was performed with cells having a passage number 11 while Delta 2 culture was done with cell passage number 8. The large deviation in lactate concentration could be related to their different cell passage numbers, as previously mentioned.^59^ The latter work shows that Delta pools demonstrated increased variability with increased culture age. The lactate time courses of the Beta and WuTL pools are tracked throughout culture, even in the case where a sudden lactate re‐production occurs after 10 dpi (WuTL) (Figure 4d). This increase lags the real value presumably because, in the absence of measured lactate values, online signals like pH and base (that are known to relate to lactate changes) did not show a marked change in response to the lactate accumulation (pH went from 6.96 at 10 dpi to 6.99 at 11 dpi to 6.97 at 12 dpi and base addition was never triggered as pH values stayed within the allowed deadband). Figure 4e shows that cell growth profiles can be tracked across the 17‐day culture run. It is noteworthy that even though WuTL and Beta cultures had identical viable cell density profiles until 3 dpi, the Beta culture undergoes a secondary growth spurt which is consequently tracked by the model while the WuTL culture remains in a plateau phase. Similarly, for the Delta cultures, identical viable cell density profiles are observed until 3 dpi, from which Delta culture 2 enters a secondary growth phase while Delta culture 1 does not. Interestingly, both distinct profiles are captured by the model. This is important because, when taking into account final titers, it suggests that cultures that underwent secondary growth phases had lower endpoint titers. Consequently, monitoring that cell densities remain within a plateau phase during the protein production phase would be of interest. This argument can be supported by the development of a biphasic process strategy in which cells will be allowed to grow to a certain high density; then a process trigger such as lower temperature (31°C to 34°C from 37°C) or chemical addition (sodium butyrate, valeric acid)^67^, ^68^ will be introduced to keep cells in a biomass steady state while boosting the production.

**FIGURE 5:**
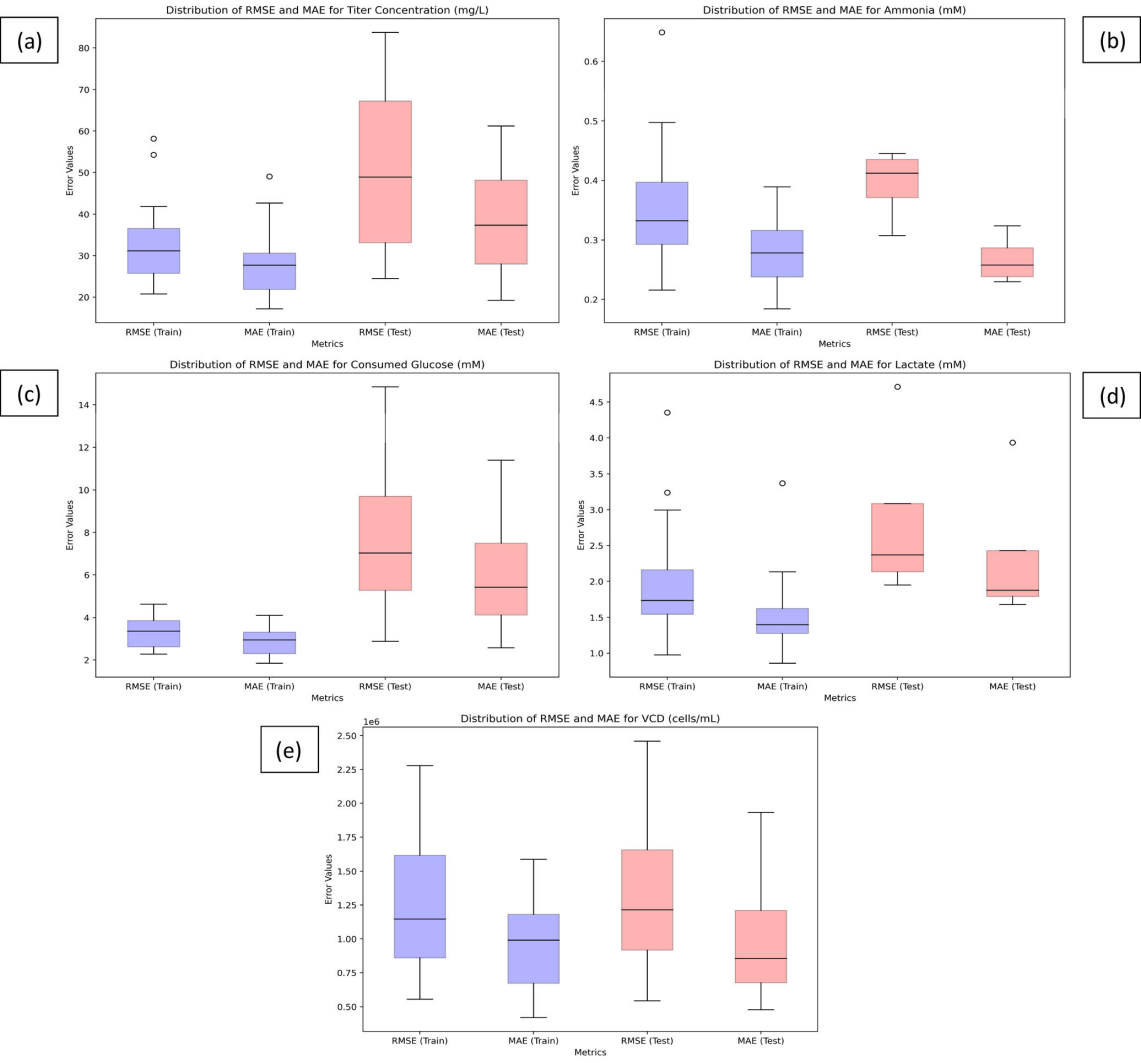
Box plot of RMSE and MAE distribution in train and test dataset for (a) Titer. (b) Ammonia concentration. (c) Cumulative glucose consumption. (d) Lactate concentration. (e) Viable cell density.

As it can be seen from Table 3, both train and test model predictions have strong linearity with respect to measured values ($R^{2}$≥0.9). The lowest $R^{2}$ values in the test set correspond to lactate, titer and VCD. These time series profiles displayed sharp variations (sudden lactate re‐absorption or secondary growth phases) causing the model predictions to lag measured values before converging again. When detailing the normalized RMSE and MAE, which take into account the standard deviation of the datasets, it can be seen that the predicted features have normalized error metrics significantly below unity (<1). This demonstrates the applicability for the model to predict the time series variation of multiple features with highly nonlinear kinetics like cellular growth and lactate formation which can increase rapidly and then decline depending on the culture phase. The model's nRMSE and nMAE metrics for VCD, titer, and lactate are in line with similar state‐of‐the‐art applications of data driven soft sensors for time series tracking.^32^, ^33^ The standard deviation of the error metrics was observed to increase between the training and test datasets across all predicted variables (Table 3). Figure 5 presents box plots of the RMSE and MAE distributions, which show that while both error metrics and their distributions increase, the interquartile ranges generally overlap between the training and test sets for all predicted variables. This suggests that the model is not subject to overfitting. The only notable outlier in the test set occurs in the lactate prediction errors for the WuTL culture (Figure 5d). This outlier can be attributed to the culture's lactate reproduction behavior, which was not adequately forecasted on day 12. An additional observation is the increased variability in the error metrics for consumed glucose in the test set (Figure 5c). This increase is due to the overestimation of glucose consumption in the Delta 1 culture and the underestimation in the Beta culture. This suggests that incorporating information about feed addition and glucose supplementation into the model could help reduce the spread in these error metrics. While the titer error does increase (Figure 5a) in the test set, it should be noted that due to its estimation with semi‐quantitative gels, the model error remains below the method's variability. This suggests that the proposed framework can aid in monitoring recombinant protein production. Furthermore, it can be observed that the distributions of error metrics for VCD (Figure 5e) are comparable between the training and test sets further supporting the notion of low overfitting.

**FIGURE 6:**
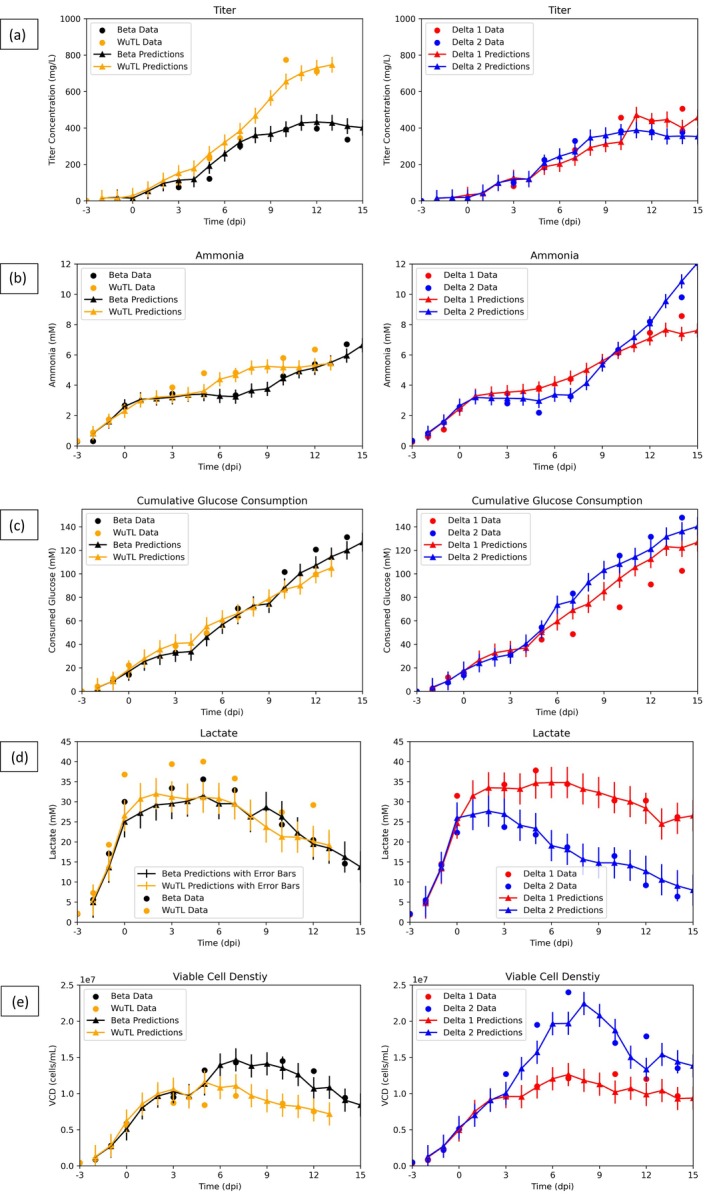
Model results for key features in the test dataset comprised four cultures (one Beta pool batch, one WuTL pool batch, and two Delta pool batches) without metabolic and titer sampling data. Continuous lines with triangle dots are everyday predictions, while circular dots are measured values. (a) Titer. (b) Ammonia. (c) Cumulative glucose consumed. (d) Lactate. (e) Viable cell density (VCD). The error bars represent the global root‐mean‐square errors of the RNN predictions based on the test dataset and the corresponding target variable.

Given that the model relies on previous day predictions and current day online data to estimate next day features and that it was shown to work reasonably well during the non‐sampling periods of the 17‐day production process, it was hypothesized that the same model without alternative training for parameter tuning could be utilized to predict metabolic data and titer profiles in the absence of such data. Consequently, the model would rely on initial feature values which function as initial guesses, sampling day cell counts, and bioreactor online data to generate predictions of ammonia, cumulative consumed glucose, lactate, and titer throughout the 17‐day process. The objective then was to evaluate if the current model has suitable potential for true soft sensing capabilities which would be of interest for hard‐to‐detect variables such as non‐antibody recombinant proteins. As it can be seen from Figure 6a,b, qualitative trends of titer and ammonia are tracked despite having no sampling day values of said features to aid in the updated prediction. Furthermore, glucose consumption (Figure 6c) is also tracked throughout the culture with only Delta 2 culture being overestimated at 14 dpi by 20 mM. Alternatively, lactate profiles (Figure 6d) for the Beta and WuTL pool are underestimated from induction until the end of the process. For the WuTL process, the lack of sampling lactate data did not allow the model to predict the sudden lactate re‐production phase (10–12 dpi). One of the reasons for this underestimation may be due to the fact that since key online data values like base addition are governed by a PID control, which is implemented with a pH deadband, indirect estimation of lactate may lag or be underestimated. One such example is the case of the WuTL and Delta 1 cultures. WuTL produces a higher peak lactate (40 to >37.8 mM) while having lower total base addition (12 to <13.3 mL). This pH deadband activation is then very connected to the lactate absorption which pushes the pH values away from the activating deadband limit. Additionally, predicting lactate re‐production in the end phase of the culture within the context of pH deadbands also becomes difficult as the previous lactate consumption coupled with the base addition during the lactate production phase necessarily drives pH values closer to the upper edges of the pH deadband. Thus, any low amounts of lactate production will not be enough to activate base addition and will generally result in pH remaining closer to the upper edges of the pH deadband. For example, in the case of the WuTL culture from 7 dpi until 12 dpi, daily average pH values are [6.96, 6.98, 6.99, 6.96, 6.99, 6.99] and total carbon dioxide sparged values increased every day from 7 to 12 dpi [4603, 4650, 4680, 5211, 5866, 6023 mL] to control the pH in the deadband (6.9–7.0). It must also be noted that global RMSE did increase for lactate prediction when compared to operating the model with sampling day values for metabolites. Similarly, although cell counts were added to the model every sampling day for prediction, nRMSE and nMAE did increase when compared to the previous results (Table 3) in which all sampling data was available to aid in feature predictions. It suggests that the measured quantities regarding lactate accumulation, glucose consumption, ammonia accumulation, and protein production aided in next day prediction of cell culture growth dynamics.

As shown in Table 4, only VCD shows a reduction in $R^{2}$ indicating slight deviation in linearity from predictions. An increase in nRMSE and nMAE can be observed for lactate, ammonia, and VCD predictions indicating slight increase in error predictions. However, despite the increase in error metrics, qualitative tracking is still possible. This suggests that the model architecture with cell count measurements (which are easy to obtain manually let alone with dedicated cell counting machines) and online data from the bioreactor is robust enough to soft sense hard‐to‐detect variables (in this case metabolic variables and protein production). This is especially interesting within the context of difficult‐to‐quantify proteins such as the SARS‐CoV‐2 spike protein since quantification is generally done through ELISA assays or semi‐quantitative SDS‐PAGE once the process is finished.^56^, ^58^ Consequently, throughout the process, no knowledge regarding the trajectory of the titer values is known, hampering decision making and slowing down proposals for process improvement since retrospective analysis of titers needs to be realized before conclusions can be drawn.

**TABLE 4:** Global RMSE, MAE, nMAE, nRMSE, $R^{2}$ metrics and their respective standard deviations for titer, ammonia, cGC (cumulative glucose consumed), lactate, VCD (viable cell density) for the test dataset without utilizing sampling day metabolic and titer data in model predictions.

	${\text{RMSE}}_{\text{test}}$	${\mathrm{MAE}}_{\text{test}}$	${R^{2}}_{\text{test}}$	${\text{nMAE}}_{\text{test}}$	${\text{nRMSE}}_{\text{test}}$	$\sigma {\text{RMSE}}_{\text{test}}$	$\sigma {\mathrm{MAE}}_{\text{test}}$
Titer	48.3	38.1	0.98	0.19	0.24	18.2	13
Ammonia	0.5	0.3	0.98	0.15	0.22	0.1	0.1
cGC	7.9	6.0	0.99	0.15	0.20	4.3	3.3
Lactate	3.9	3.4	0.95	0.36	0.41	2.1	2.1
VCD	1.60e6	1.19e6	0.94	0.24	0.33	6.4e5	5.46e6

Given that estimation of cell growth and cumulative glucose consumption rates were accurate even when no glucose consumption data was utilized in the test set, estimation of specific glucose consumption rates (qGluc) was performed. Since the model prediction represents cumulative glucose consumption up to any given day, the derivative of this output gives the glucose consumed every day (since every prediction is equally spaced in the time dimension, the derivative is calculated using the difference between consecutive datapoints such that Consumed_Glucose= ${\mathrm{cGC}}_{\left(i+1\right)}-{\mathrm{cGC}}_{\left(i\right)}$. Given the VCD predictions, the integral of viable cell concentration (IVCC) can be estimated through the trapezoid rule of numerical integration. Consequently, differentiating these IVCC values allows for the estimation of ΔIVCC (ΔIVCC = ${\text{IVCC}}_{\left(i+1\right)}-{\text{IVCC}}_{\left(i\right)}$). If the consumed glucose estimated at every time point is then divided by the ΔIVCC at every point in time $\left({\text{ΔIVCC}}_{i}\right)$, predicted specific glucose consumption rates $\left({\text{qGluc}}_{\text{pred}}\right)$ can be calculated.

$${\text{qGluc}}_{\text{pred}}=\frac{\text{Consumed}\_{\text{Glucose}}_{i}}{\Delta {\text{IVCC}}_{i}}$$

The ground data of glucose consumed between sampling days $\left(\text{Consumed}\_{\text{Glucose}}_{k}\right)$ can be divided by the ΔIVCC_*k*_ (change in IVCC between sampling days), then specific glucose consumption rates can be estimated. The subscript *k* indicates the ground data at sampling day *k*.

$${\text{qGluc}}_{\text{Ground}\_\text{Truth}}=\frac{\text{Consumed}\_{\text{Glucose}}_{k}}{\Delta {\text{IVCC}}_{k}}$$

**FIGURE 7:**
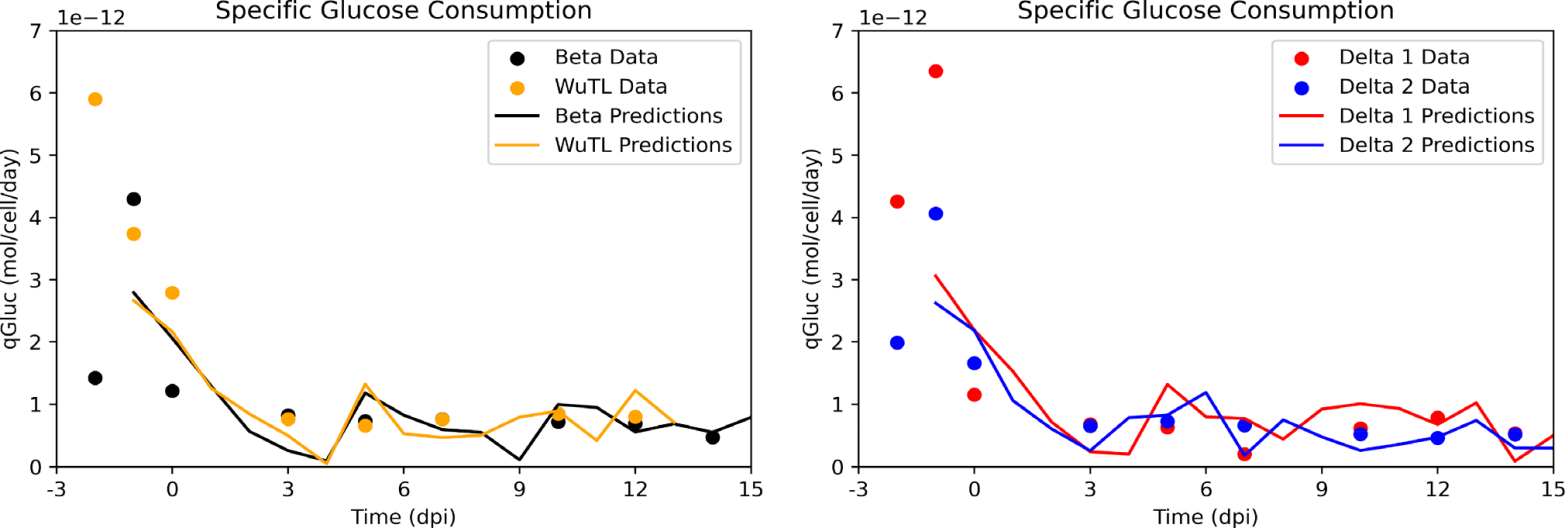
Specific glucose consumption rate (qGluc) estimation for Test dataset comprised of 4 cultures (1 Beta pool batch, 1 WuTL pool batch, and 2 Delta pool batches). Continuous lines are everyday predictions while dots are measured values.

In Figure 7, it is possible to see an overlay between the estimated specific glucose consumption rates and the predicted specific glucose consumption rates. For the 4 test cases, qualitative tracking of specific glucose consumption rates is possible, showing a fast decrease from −1 to 0 dpi, which represents the end of the growth phase and then near constant qGluc after temperature downshift. This qualitative tracking of specific glucose consumption rates (qGluc) is important, as knowledge regarding the specific consumption represents knowledge regarding its internal metabolic state.^69^, ^70^ Additionally, having 1 day ahead predictions of specific glucose consumption rates represents a pseudo‐online opportunity for nutrient control which has been tried utilizing various approaches (Raman spectroscopy, optical probes, oxygen monitoring).^71^, ^72^, ^73^, ^74^ Here, glucose concentrations inside the bioreactor can be adjusted based on the prediction of specific glucose consumption rates such that glucose levels within the bioreactor are maintained within a given set point. Such monitoring and control strategies have shown promise in reducing product glycation.^75^ Given the qualitative accuracy of tracking specific glucose consumption rates, a similar approach may be taken to track specific lactate production/consumption rates, specific ammonia accumulation, growth rates, and specific protein production rate. Additionally, since it was observed that indirect measurements could be learned (qGluc), it can be postulated that by adding total cell density (TCD) prediction into the model, viability (viability = VCD/TCD) can be indirectly monitored throughout the manufacturing process.

It is also worth noting that the online data available in this case study is routine online data such as temperature, pH, DO, oxygen injection, and carbon dioxide addition. Thus, it stands to reason that as more biologically relevant online sensor data is added to the processes and consequently the model, better predictive capabilities can be achieved. Such sensor data that could be utilized is oxygen uptake rates (OUR) and bio‐capacitance signals.^1^ OUR has been known to relate both to cellular growth and metabolic activity as peak antibody production has been associated with increased oxidative metabolism which, in turn, means an increased oxygen demand.^76^, ^77^, ^78^, ^79^, ^80^, ^81^, ^82^ Alternatively, bio‐capacitance signals have been observed to not only relate to total viable cells within the culture but also to total biovolume as the capacitance signal is dependent on the volume of each cell.^83^, ^84^, ^85^ This is key as cellular diameter has been observed to increase during a culture run^86^ and consequently changes in cellular volume are expected. Cell volume also encodes information regarding cellular phase and recombinant protein production activity.^87^, ^88^ Given the close relationship between bio‐capacitance and cellular volume, further improvements to the model's predictive capabilities could be made. Notably, sensor fusion (biocapacities, OUR and fluorescence data) coupled with machine learning techniques have demonstrated strong monitoring capacity of relevant features such as cellular viability, cellular density, metabolites, and viral yield^89^ further underscoring the potential for improved process forecasting with the proposed model when coupled with process analytical technologies (PAT). Unlike mechanistic approaches, the proposed RNN modeling strategy can incorporate dynamically evolving parameters like temperature, pH, dissolved oxygen content, base addition, and gassing profiles to improve feature prediction. These bioreactor values, which are not easily modeled with simple dynamic equations, hold valuable information about cell culture behavior, as the control loops are directly linked to cell culture activity (e.g., lactate accumulation driving base addition through pH drops and oxygen requirements influencing oxygen supplementation via DO drops). Another major advantage of this data‐driven method, compared to mechanistic or hybrid models, is that the pre‐trained RNN can be readily applied by non‐experts, as it doesn't require knowledge of boundary conditions or metabolic networks. This makes it easily transferable to production processes without additional training for operators. Moreover, the RNN's ability to capture internal temporal dependencies within the system enhances feature predictions, offering an advantage over models that assume independent observations for each prediction. However, a key downside of this approach is its reliance on high‐quality process data. Its application to new, previously unseen processes or parameter variations not represented in the training set may lead to poor performance. Furthermore, RNN models can be computationally intensive, requiring large datasets and significant training time, which may limit their use in data‐scarce environments. Additionally, unlike mechanistic models, RNNs are less interpretable, which can complicate understanding of the underlying processes and limit their use in regulated environments. In contrast, hybrid models, which combine mechanistic insights with data‐driven techniques, may offer a more robust and interpretable solution for handling a broader range of process variations. To address the generalization challenge, one approach is to couple the RNN model with a system of differential equations that describe the dynamics of the process. For example, since specific glucose consumption rates can be reasonably predicted, it is plausible that other specific rates, such as specific ammonia production, specific lactate production, and specific protein production, could also be estimated. These specific rates can then be used as parameters in dynamic equations to construct a more robust prediction ensemble. This approach has been explored with data‐driven regressors that assume independent observations,^41^ and it stands to reason that utilizing models that leverage the temporal dependencies in the data for specific rate predictions could prove to be a worthwhile strategy.

It is important to note that this method requires historical data to train the model effectively. This data can typically be acquired during the process characterization phase of late‐stage cell culture processes, particularly when producing novel therapeutics for approval.^90^ Scale‐down models, which demonstrate behaviors comparable to their GMP manufacturing counterparts, provide a valuable platform for generating large datasets that can be used to train the base model before a fine tuning phase using large‐scale GMP bioreactor data; such a transfer learning approach could prove feasible within the biomanufacturing industry framework. Furthermore, within platform processes, it is reasonable to assume that initial model base development can leverage data from similar cell lines or manufacturing processes. This approach allows for the construction of a base model framework, which can then be iteratively finetuned with relevant data as it becomes available. Importantly, to avoid pH sensor drift issues, the model could incorporate both online and offline pH sensor measurements as well as the delta offset between them to build redundancy and avoid the negative impact of possible sensor drift. In data‐scarce environments, the use of synthetic data has also shown promise.^52^ Mechanistic models can generate culture profiles, which can serve as an initial data source for model training. However, it is worth mentioning that, with the presented approach, online data from gassing profiles, pH, and base values have proven to be valuable in feature prediction. These real‐time measurements may be more challenging to simulate alongside cell culture kinetics in mechanistic models, further underscoring the advantage of incorporating actual process data in this data‐driven approach.

## 4 CONCLUSION

The proposed soft sensor architecture can accurately (nRMSE, nMAE are below unity and $R^{2}$ ≥ 0.9 for all features) predict product titer, total glucose consumption, ammonia, lactate, and viable cell densities, and for a long‐term process (17 days) with ground sampling day data only available every other day or every two days to aid in next iteration predictions. To counter the lack of everyday sampling data, daily online data was also utilized into the model. The online data, although harder to directly interpret, still contains relevant information about culture phase (temperature downshifts denote process induced decrease in cellular growth to prime the process for protein production). In terms of PID controllers, it indirectly contains information about oxygen demand (DO, total oxygen sparge) and lactate metabolism (pH, base addition, total carbon dioxide addition). Interestingly, once the same model was applied in a test case where no ground sampling day data was given for titers, glucose consumption, ammonia, and lactate throughout the 17‐day culture process, it was determined that the model was still effectively able to soft sense these hard‐to‐measure features (nRMSE and nMAE below unity and $R^{2}$ ≥ 0.9 for all features). This is especially interesting in processes where the recombinant protein in question can be difficult to measure as is the case of the trimeric SARS‐CoV‐2 spike protein. In such cases, having a qualitative tracking of feature evolution can be of value. This was possible by considering the bioreactor data that is routinely available in all commercial bioreactor systems. Additionally, qualitative tracking of specific glucose consumption rates (qGluc) was enabled with the proposed method allowing for the possibilities of tight glucose control inside bioreactors by relying on the one day ahead specific glucose consumption rates predictions and adjusting glucose addition to keep overall glucose concentration near a given setpoint. With this knowledge, it stands to reason that the proposed soft sensor can gain from further use of process analytical technologies (PAT) such as off‐gas analyzers, bio‐capacitance, and Raman spectroscopy signals from which biologically relevant signals can be related to the discretely measured features.

## AUTHOR CONTRIBUTIONS

**Sebastian Juan Reyes**: Data curation; data analysis; methodology; writing‐original draft preparation. **Robert Voyer, Yves Durocher**: Supervision; writing–review. **Olivier Henry**: Supervision; writing–review and editing. **Phuong Lan Pham**: Experimental conceptualization; Supervision; writing–review and editing.

## CONFLICT OF INTEREST STATEMENT

The authors declare no conflict of interest.

## Supporting information


**Figure S1.** Raw training data of the titer profiles colored by pool type.
**Figure S2.** Raw training data of the lactate profiles colored by pool type.
**Figure S3.** Raw training data of the ammonia profiles colored by pool type.
**Figure S4.** Raw training data of the consumed glucose profiles colored by pool type.
**Figure S5.** Raw training data of the viable cell density (VCD) profiles colored by pool type.
**Figure S6.** Prediction and true values overlay of titer for each pool type and its associated percentage error over time in the train dataset. Percentage error of titers are shown from 3dpi onwards as induction and protein production begins after 0 dpi.
**Figure S7.** Prediction and true values overlay of lactate for each pool type and its associated percentage error over time in the train dataset.
**Figure S8.** Prediction and true values overlay of ammonia for each pool type and its associated percentage error over time in the train dataset.
**Figure S9.** Prediction and true values overlay of consumed glucose for each pool type and its associated percentage error over time in the train dataset.
**Figure S10.** Prediction and true values overlay of VCD for each pool type and its associated percentage error over time in the train dataset.

## Data Availability

The data that support the findings of this study are available from the corresponding author upon reasonable request.
