# Supplementary material for: A recurrent neural network for soft sensor development using CHO stable pools in fed‐batch process for SARS‐CoV‐2 spike protein production as a vaccine antigen
Source: Biotechnol Prog. 2025 Jun 2;41(5):e70046. doi: 10.1002/btpr.70046 (PMC12531939; doi:10.1002/btpr.70046)
Supplement: Supplementary file 1 — Figure S1. Raw training data of the titer profiles colored by pool type. Figure S2. Raw training data of the lactate profiles colored by pool type. Figure S3. Raw training data of the ammonia profiles colored by pool type. Figure S4. Raw training data of the consumed glucose profiles colored by pool type. Figure S5. Raw training data of the viable cell density (VCD) profiles colored by pool type. Figure S6. Prediction and true values overlay of titer for each pool type and its associated percentage error over time in the train dataset. Percentage error of titers are shown from 3dpi onwards as induction and protein production begins after 0 dpi. Figure S7. Prediction and true values overlay of lactate for each pool type and its associated percentage error over time in the train dataset. Figure S8. Prediction and true values overlay of ammonia for each pool type and its associated percentage error over time in the train dataset. Figure S9. Prediction and true values overlay of consumed glucose for each pool type and its associated percentage error over time in the train dataset. Figure S10. Prediction and true values overlay of VCD for each pool type and its associated percentage error over time in the train dataset. [file BTPR-41-e70046-s001.docx]

**ANNEX**


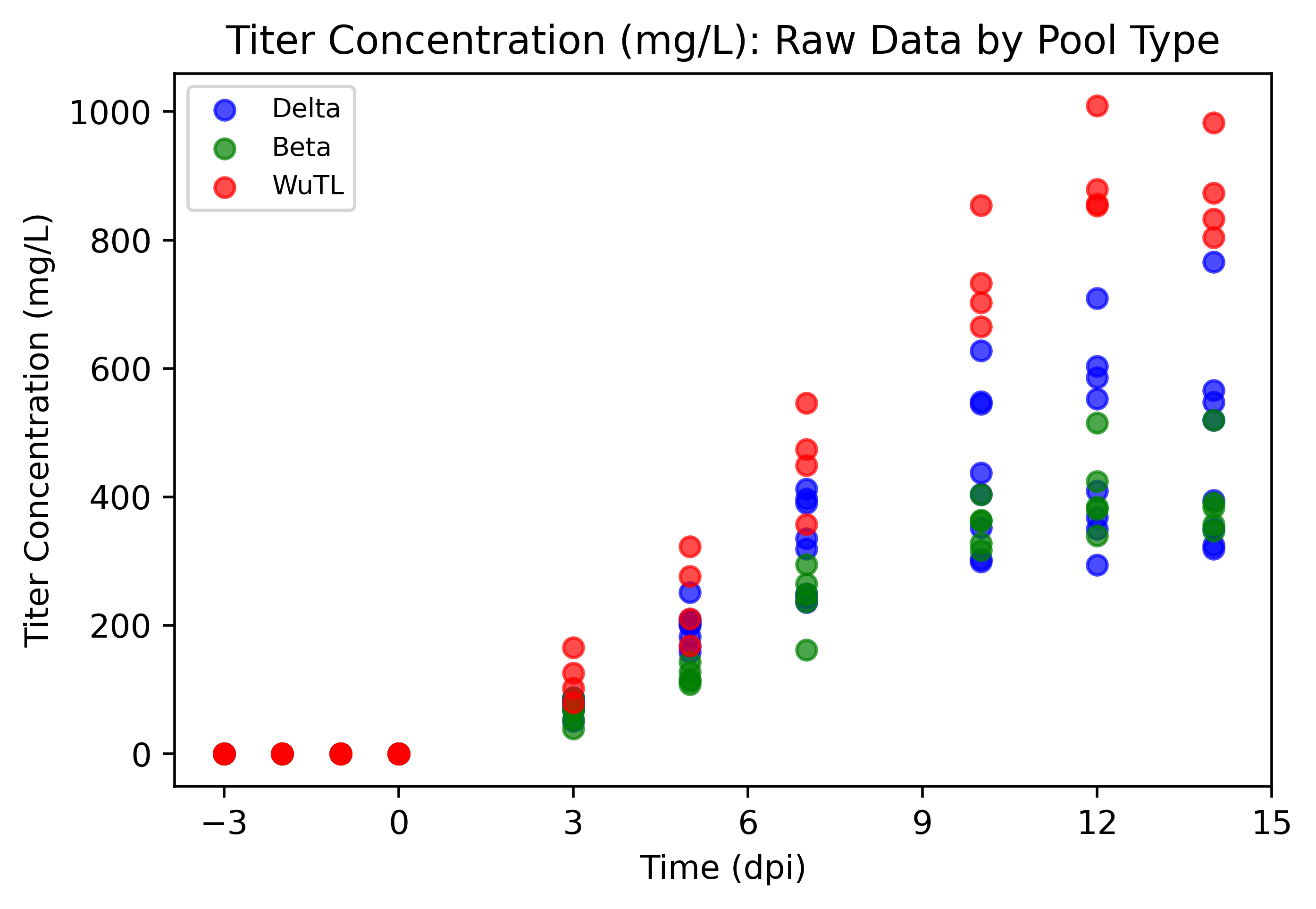


**Figure 1S. Raw training data of the titer profiles colored by pool type.**


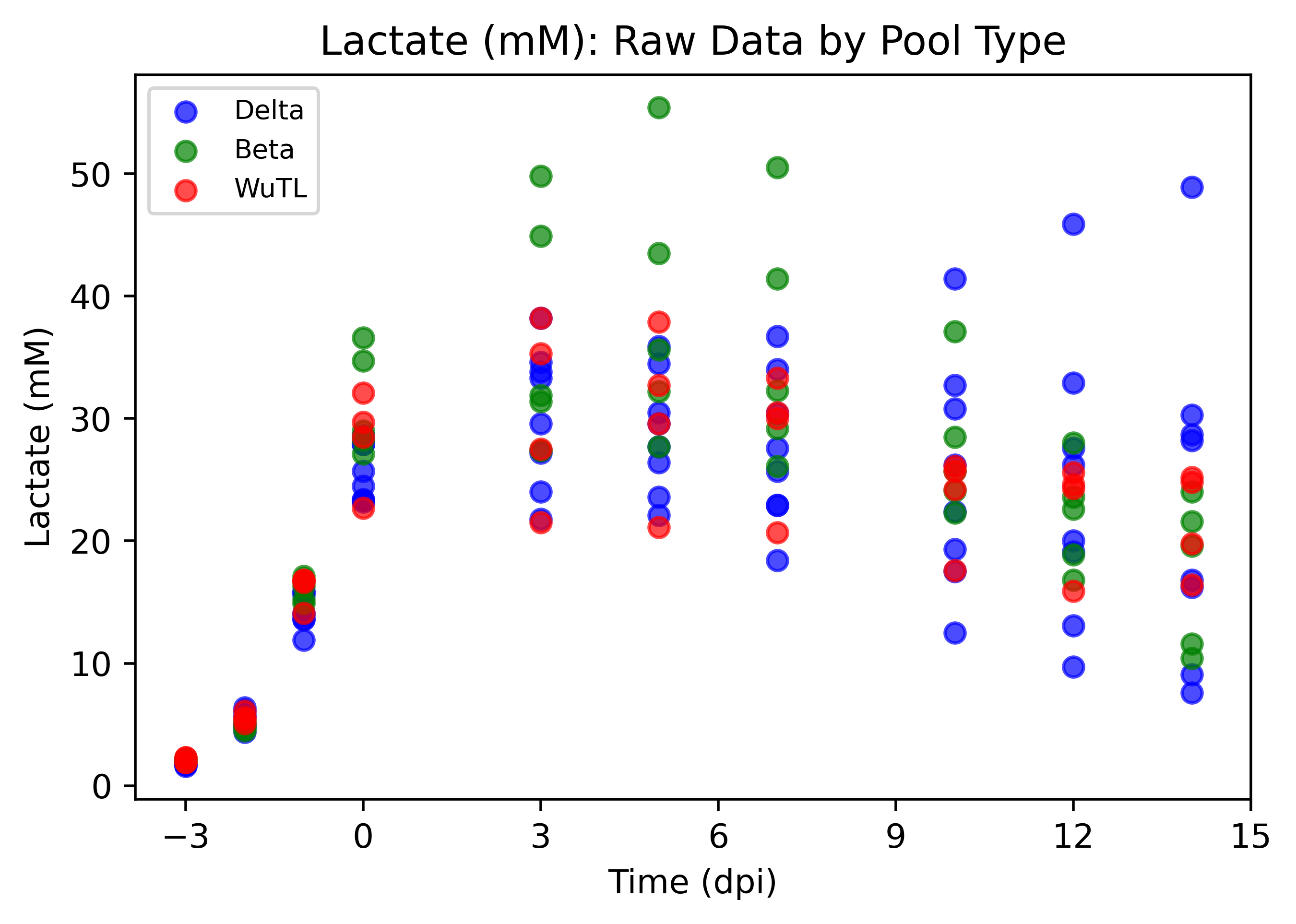


**Figure 2S. Raw training data of the lactate profiles colored by pool type.**


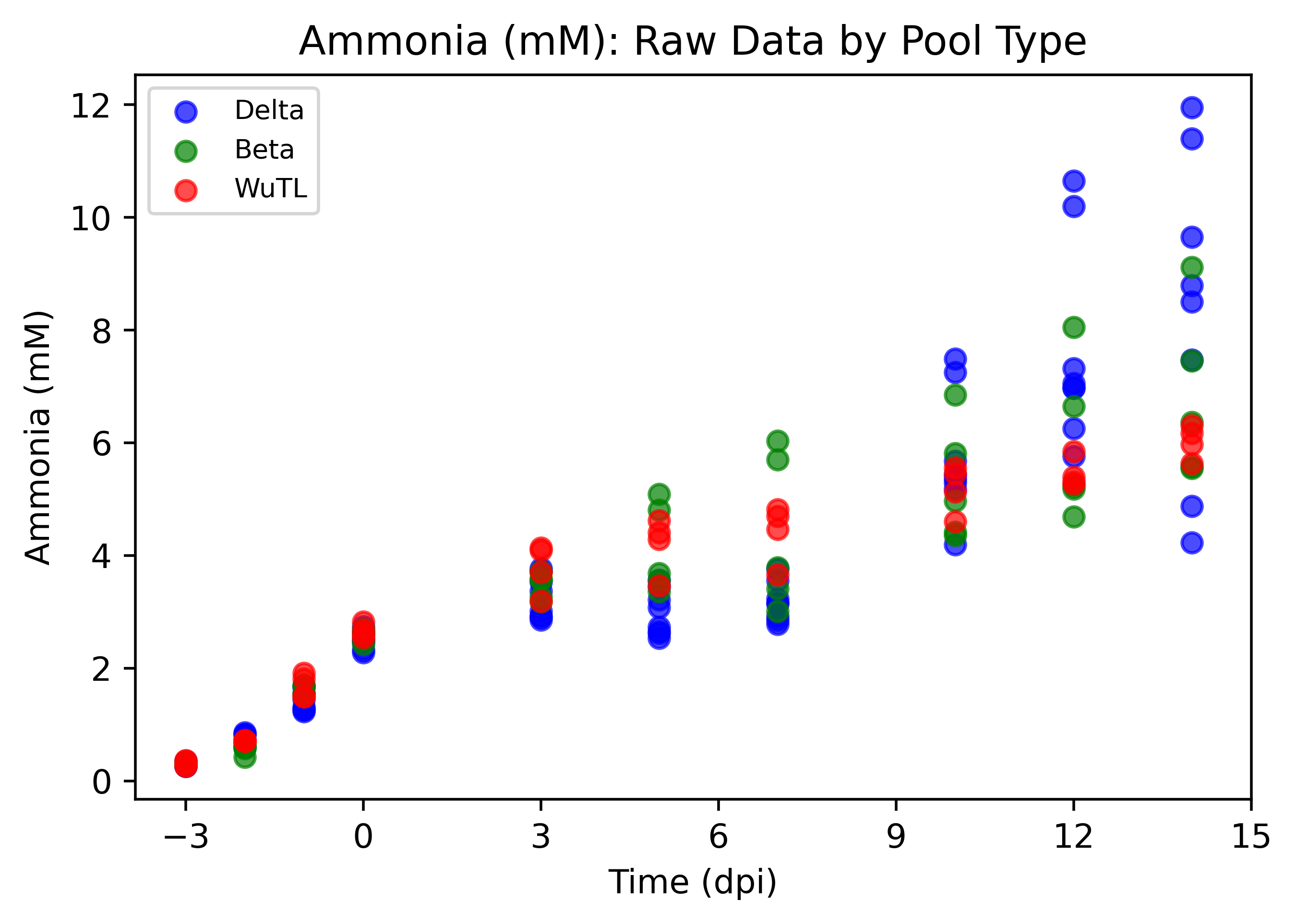


**Figure 3S. Raw training data of the ammonia profiles colored by pool type.**


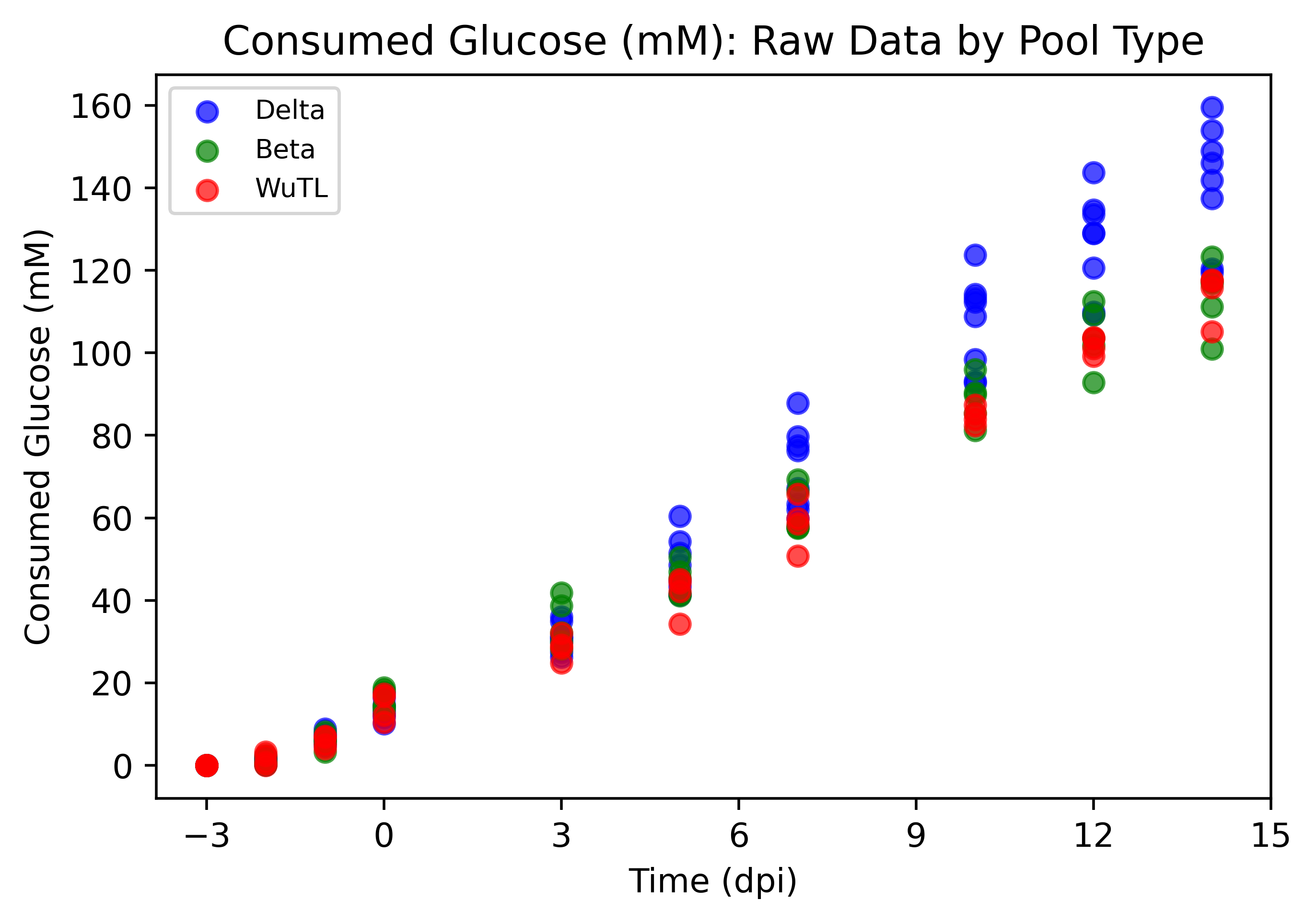


**Figure 4S. Raw training data of the consumed glucose profiles colored by pool type.**


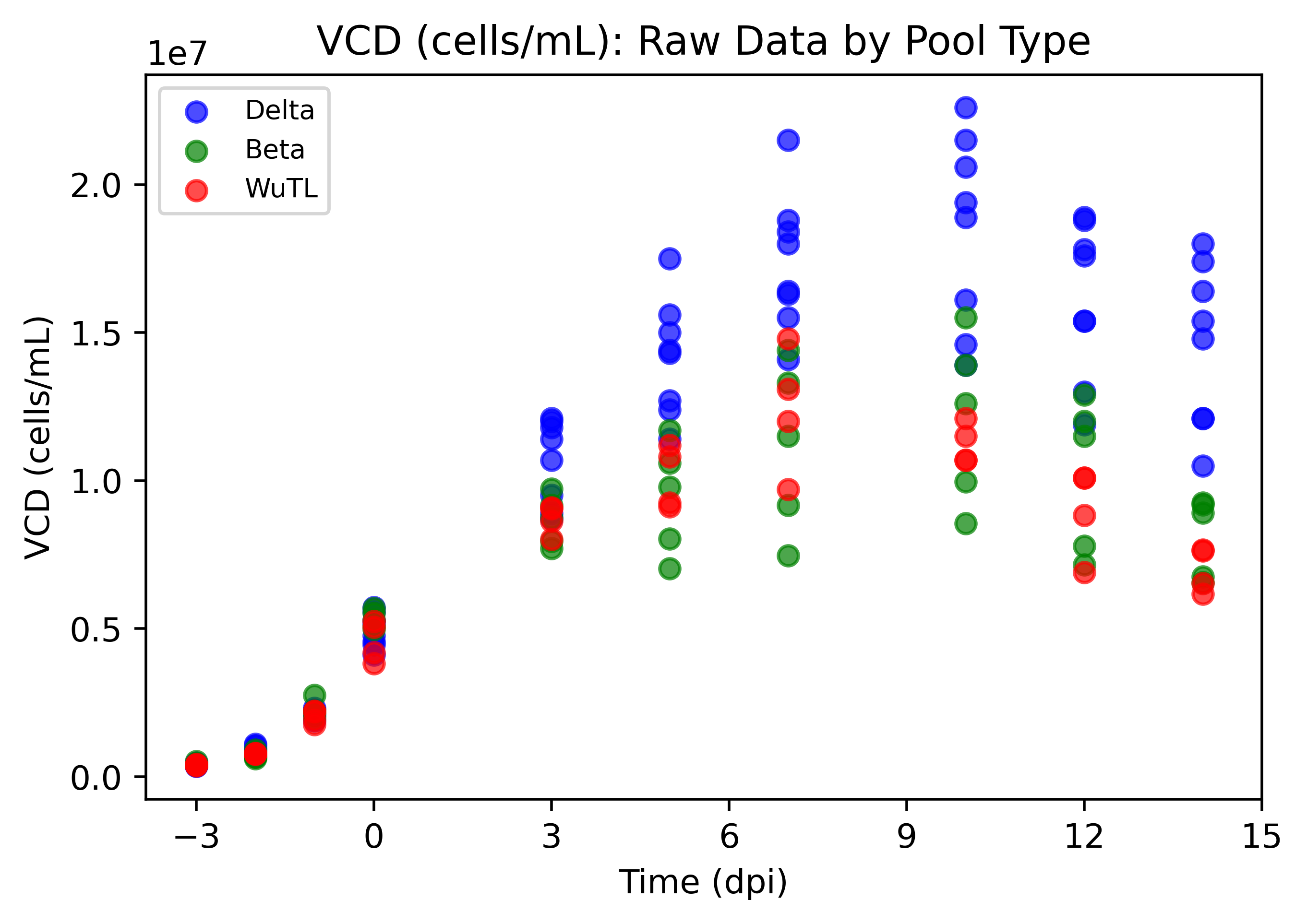


**Figure 5S. Raw training data of the viable cell density (VCD) profiles colored by pool type.**

**
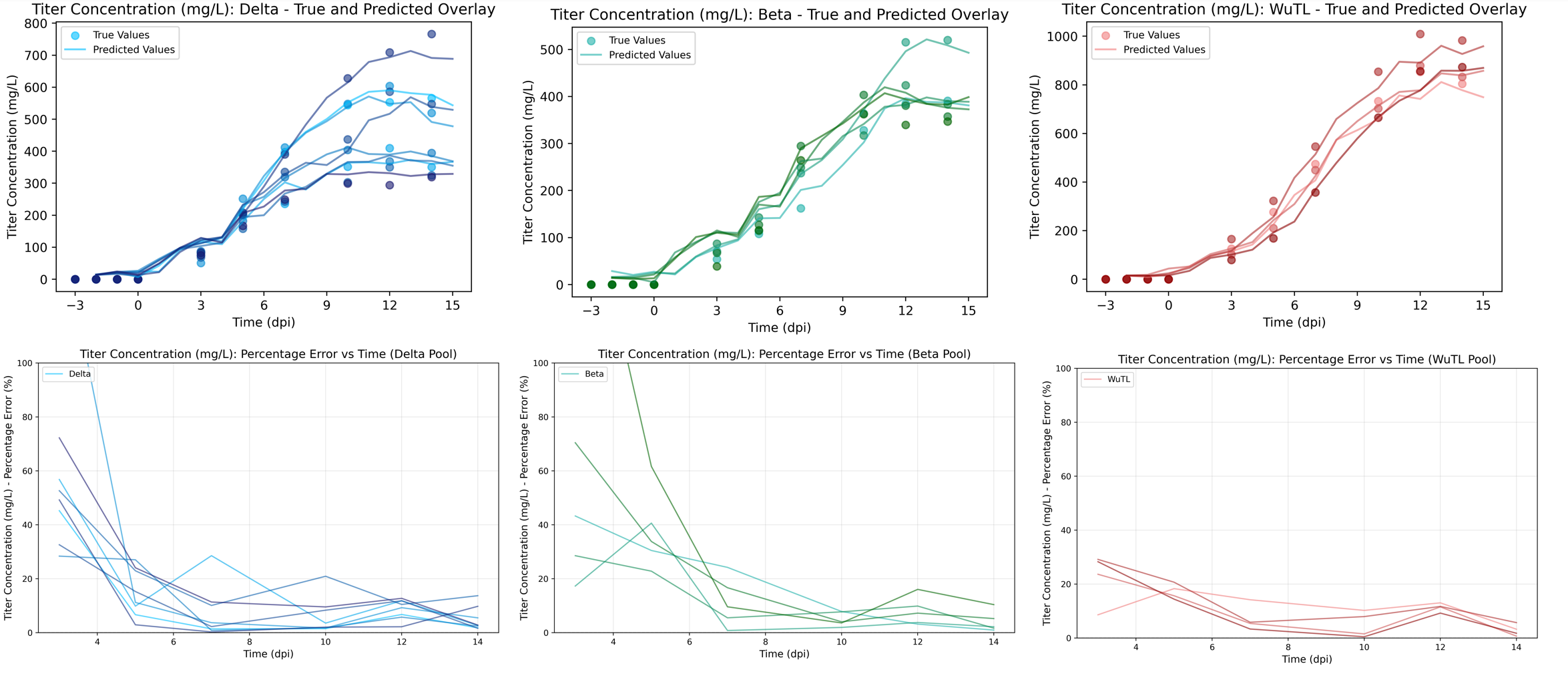
**

**Figure 6S. Prediction and true values overlay of titer for each pool type and its associated percentage error over time in the train dataset. Percentage error of titers are shown from 3dpi onwards as induction and protein production begins after 0 dpi.**

**
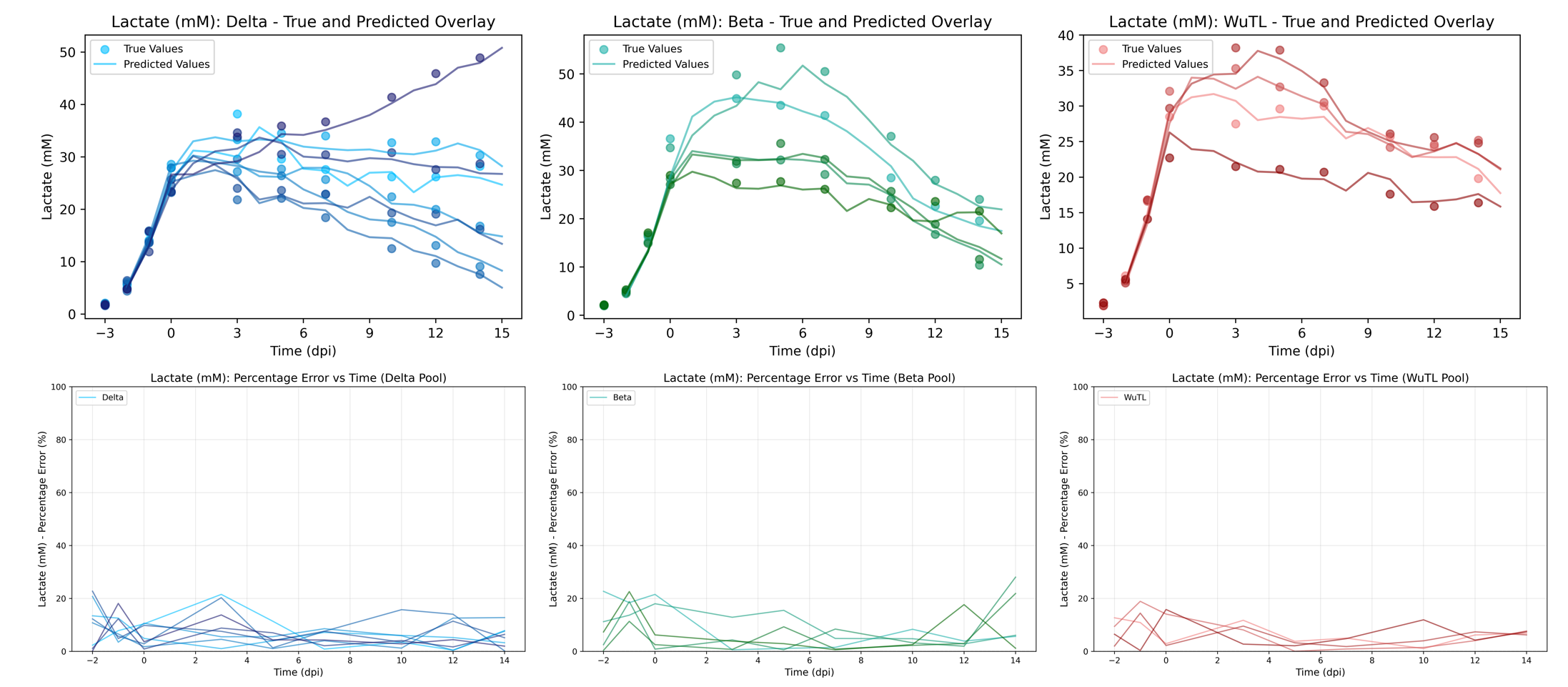
**

**Figure 7S. Prediction and true values overlay of lactate for each pool type and its associated percentage error over time in the train dataset.**

**
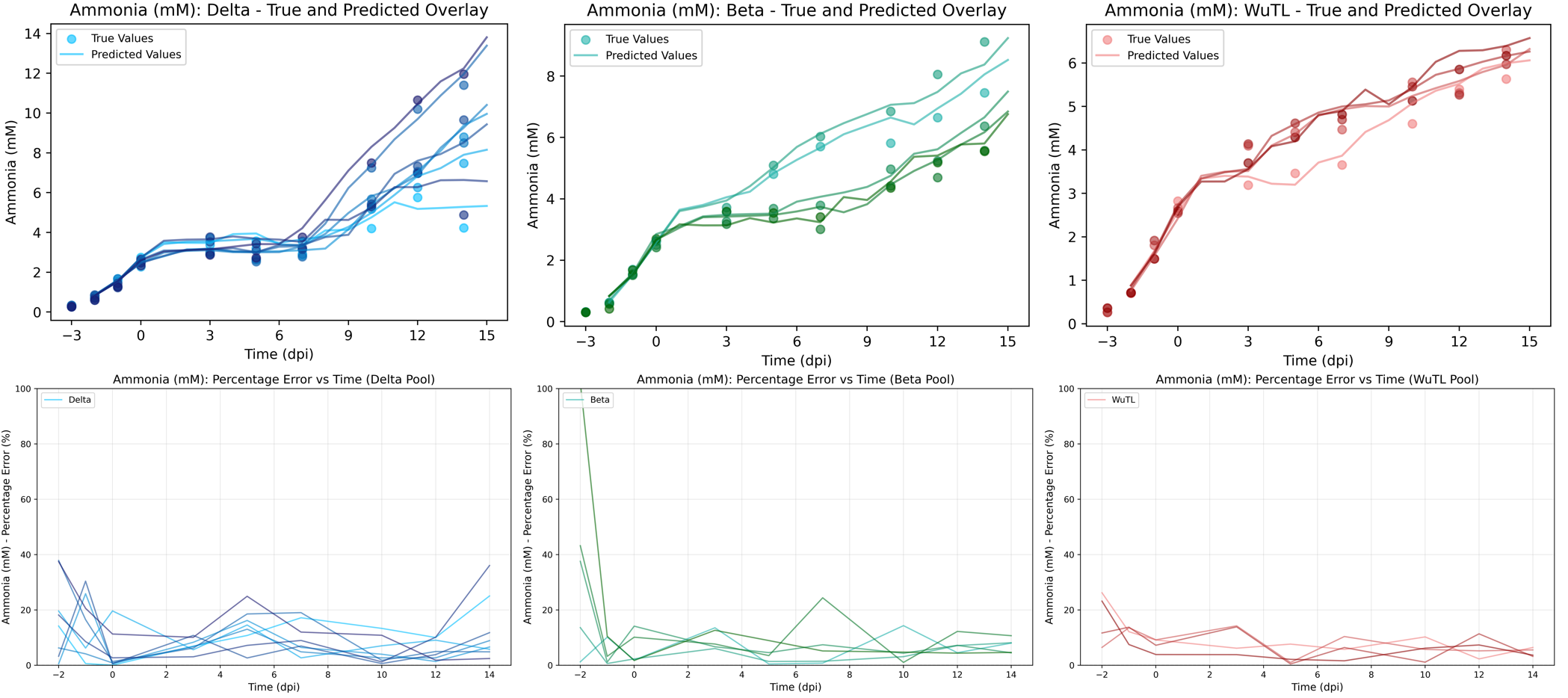
**

**Figure 8S. Prediction and true values overlay of ammonia for each pool type and its associated percentage error over time in the train dataset.**

**
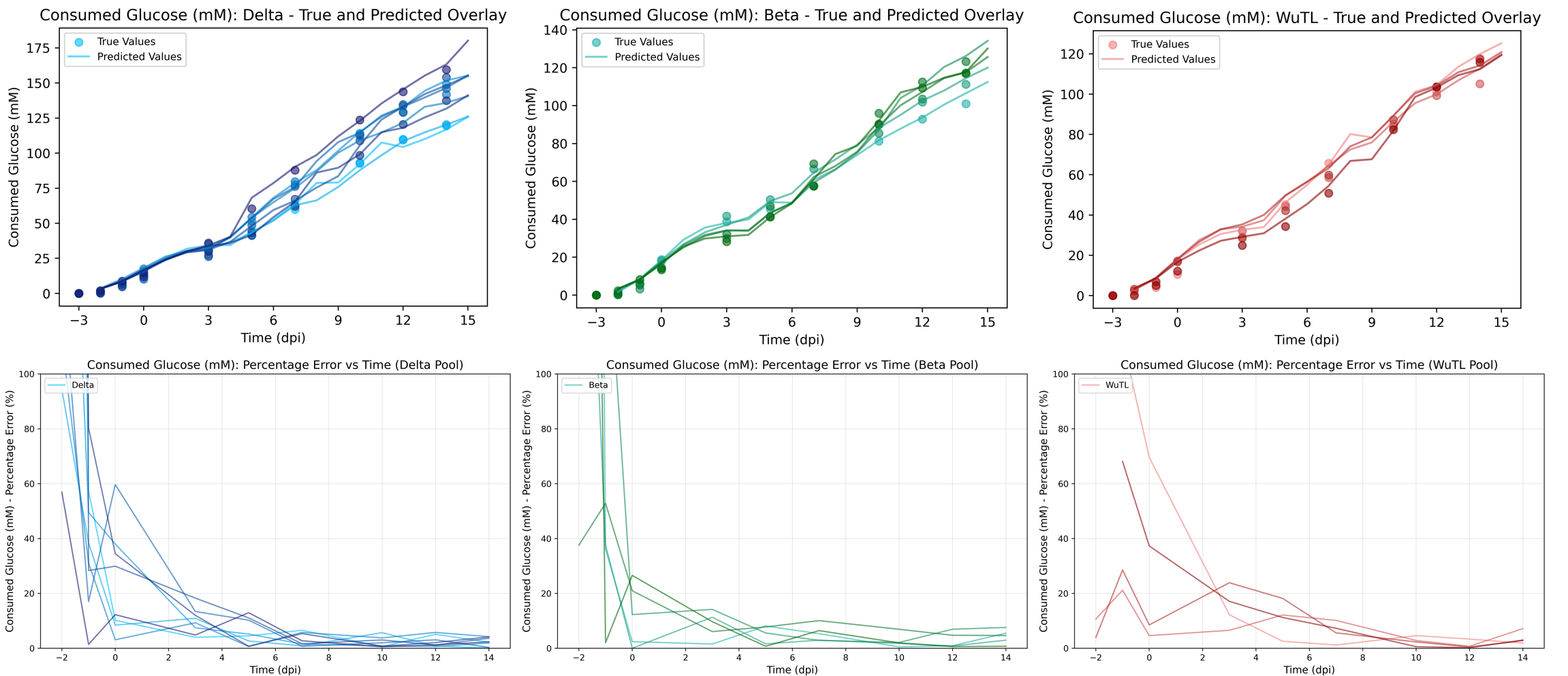
**

**Figure 9S. Prediction and true values overlay of consumed glucose for each pool type and its associated percentage error over time in the train dataset.**

**
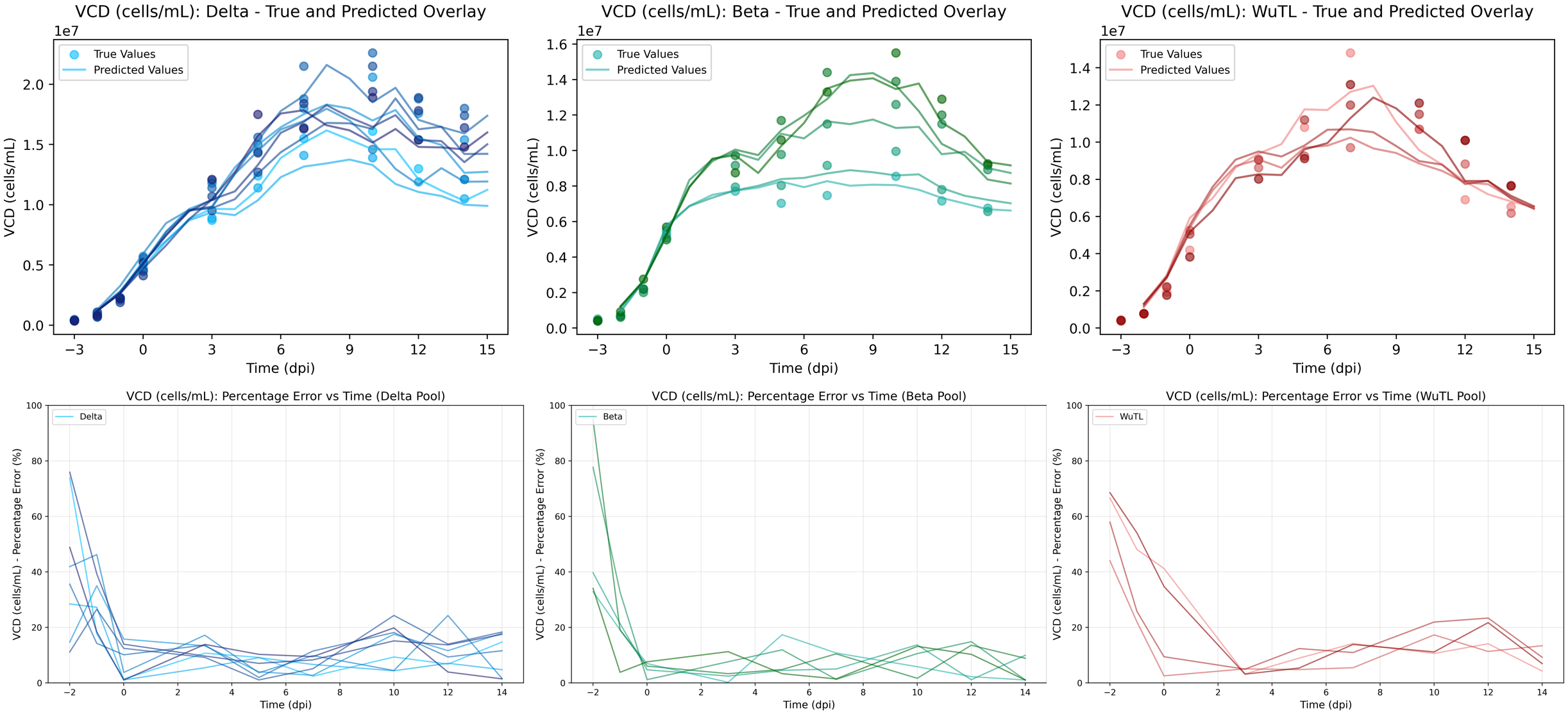
**

**Figure 10S. Prediction and true values overlay of VCD for each pool type and its associated percentage error over time in the train dataset.**
